# Proteomic analysis across aged tissues reveals distinct signatures and the crucial involvement of midgut barrier function in the regulation of aging

**DOI:** 10.1111/acel.14344

**Published:** 2024-09-25

**Authors:** Congying Zhang, Jinlong Wang, Tianzhao Yao, Jiaxin Hu, Feifei Sun, Chunlu Feng, Zhendong Sun, Yuzhuo Shao, Zhu Wang, Jiarui Wu, Yunpeng Huang

**Affiliations:** ^1^ Key Laboratory of Systems Health Science of Zhejiang Province, School of Life Science, Hangzhou Institute for Advanced Study University of Chinese Academy of Sciences Hangzhou China; ^2^ Key Laboratory of Systems Biology, Hangzhou Institute for Advanced Study University of Chinese Academy of Sciences, Chinese Academy of Sciences Hangzhou China

**Keywords:** aging, *Drosophila*, gut barrier, insulin receptor, proteome

## Abstract

The process of aging is a natural phenomenon characterized by gradual deterioration in biological functions and systemic homeostasis, which can be modulated by both genetic and environmental factors. Numerous investigations conducted on model organisms, including nematodes, flies, and mice, have elucidated several pivotal aging pathways, such as insulin signaling and AMPK signaling. However, it remains uncertain whether the regulation of the aging process is uniform or diverse across different tissues and whether manipulating the same aging factor can result in consistent outcomes in various tissues. In this study, we utilize the *Drosophila* organism to investigate tissue‐specific proteome signatures during the aging process. Although distinct proteins undergo changes in aged tissues, certain common altered functional networks are constituently identified across different tissues, including the decline of the mitochondrial ribosomal network, autophagic network, and anti‐ROS defense networks. Furthermore, downregulation of insulin receptor (InR) in the midguts, muscle, and central nervous system (CNS) of flies leads to a significant extension in fly lifespans. Notably, despite manipulating the same aging gene InR, diverse alterations in proteins are observed across different tissues. Importantly, knockdown of InR in the midguts leads to a distinct proteome compared with other tissues, resulting in enhanced actin nucleation and glutathione metabolism, while attenuating age‐related elevation of serine proteases. Consequently, knockdown of InR results in rejuvenation of the integrity of the midgut barrier and augmentation of anti‐ROS defense capabilities. Our findings suggest that the barrier function of the midgut plays a pivotal role in defending against aging, underscoring the paramount importance of maintaining optimal gut physiology to effectively delay the aging process. Moreover, when considering age‐related changes across various tissues, it is more reasonable to identify functional networks rather than focusing solely on individual proteins.

## Graphical abstract

Proteomic analysis reveals that instead of specific proteins, different tissues exhibit common dysregulated functional networks. Specifically, the integrity of the midgut barrier function is compromised during aging, which can be restored through the downregulation of the insulin receptor (InR).

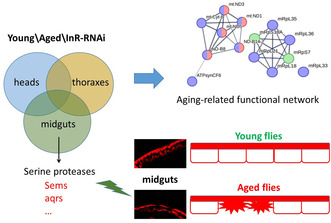

**Abbreviations**
Agt4b: Autophagy‐related 4b
AMPK: Adenosine monophosphate‐activated kinase
Aprt: Adenine phosphoribosyltransferase
aqrs: Aquarius
BDSC: Bloomington Drosophila Stock Center
ben: Bendless
Bet5: Blocked early in transport 5
bin3: Bicoid‐interacting protein 3
btv: Beethoven
c(3)G: Crossover suppressor on 3 of Gowen
Cdc16: Cell division cycle 16
CNS: Central nervous system
Cog6: Component of oligomeric golgi Golgi complex 6
Cpr65Av: Cuticular protein 65Av
ctp: Cut up
CtsG: Cathepsin G
DGRC: Drosophila Genetic Resource Center
Dhps: Deoxyhypusine synthase
EMC6: ER membrane protein complex subunit 6
fdl: Fused lobes
gek: Genghis khan
Gk: Glycerol kinase
H&E: Hematoxylin and eosin
Hsp22: Heat shock protein 22
Imd: Immune deficiency
Indy: I’m not dead yet
InR: Insulin receptor
IP3K2: Inositol 1,4,5‐triphosphate kinase 2
laza: Lazaro
Lsp1beta: Larval serum protein 1 beta
MBR: Match between runs
MMPs: Matrix metalloproteinases
mRpL53: Mitochondrial ribosomal protein L53
mRpS31: Mitochondrial ribosomal protein S31
Ms: Myosuppressin
mt: C‐I: Mitochondrial respiratory complex I
mth: Methuselah
mTOR: Mechanistic target of rapamycin
Naa20A: N(alpha)‐acetyltransferase 20 A
nAChRbeta3: Nicotinic Acetylcholine Receptor beta3
Nepl18: Neprilysin‐like 18
Npc2a: Niemann‐Pick type C‐2a
Obp44a: Odorant‐binding protein 44a
PCA: Principal component analysis
Pdf: Pigment‐dispersing factor
PGRPs: Peptidoglycan recognition proteins
PH3: Phosphor‐Histone3
PPI: Protein‐protein interaction
Rab3‐GAP: Rab3 GTPase activating protein
Rbp6: RNA‐binding protein 6
RCC: Respiratory chain complex
Rocla: Regulator of cullins 1a
ROS: Reactive oxygen species
Sems: Serine protease Seminase
SIRT1: Sirtuin 1
Snx21: Sorting nexin 21
SP: Sex peptide
Sps2: Selenophosphate synthetase 2
Srxn: Sulfiredoxin
TMPRSS2: Transmembrane protease, serine 2
TMRE: Tetramethylrhodamine ethyl ester
TpnC41C: Troponin C at 41C
Trs31: TRAPP subunit 31
Tsp42Ea: Tetraspanin 42Ea
Tsp42Ed: Tetraspanin 42Ed
Vps13D: Vacuolar protein sorting 13D
WT: wild‐type

## 1 INTRODUCTION

Aging is a natural biological process characterized by the gradual deterioration of physiological integrity and homeostasis (Singh et al., 2019), resulting in diminished fitness, functional decline, and susceptibility to mortality (López‐Otín et al., 2013). Although the progressive accumulation of cellular damage is proposed as a prevailing factor, the physiological origins of aging remain enigmatic (López‐Otín et al., 2013). In addition, aging is implicated in the pathogenesis of various human diseases, encompassing cardiovascular disorders, malignancies, and neurodegenerative diseases (Singh et al., 2019). Therefore, revealing the underlying mechanisms of aging and developing effective approaches to intervene in the aging process is crucial for promoting healthy aging and intervening in aging‐related diseases.

Recently, emerging evidence from animal models has shed light on the role of genetic factors in controlling aging. These include genes involved in nutrient sensing pathways such as insulin/insulin receptor (InR) signaling, mechanistic target of rapamycin (mTOR), adenosine monophosphate‐activated kinase (AMPK), and Sirtuin 1 (SIRT1) (Singh et al., 2019; Yu et al., 2021). Additionally, factors related to proteostasis regulation also contribute to aging regulation, encompassing regulators of the autophagy and ubiquitin‐proteasome pathway, as well as protein chaperones (Korovila et al., 2017). Furthermore, mitochondrial homeostasis regulators also play a crucial role in the aging process (Bratic & Larsson, 2013). Other contributing factors, including stem cell exhaustion, intercellular communication (Ribeiro‐Rodrigues et al., 2023), and genomic stability regulators (Vijg & Suh, 2013), and so forth, are also encompassed.

The InR gene, a homolog of *Caenorhabditis elegans* daf‐2, detects insulin signaling and exerts influence on the aging process in flies and mice. Modulating the activity of insulin\InR signaling can decelerate aging progression and extend lifespan in *C. elegans*, flies, and mice (Blüher et al., 2003; Kenyon et al., 1993; Selman et al., 2008; Tatar et al., 2001). Low activity of IGF signaling and IIS pathway also is associated with a longer lifespan of human, indicating that the anti‐aging effects of insulin/InR signaling are evolutionally conserved (Franceschi et al., 2005). Moreover, targeting the IGF‐1 receptor in the late‐life stage has been demonstrated to significantly prolong mouse lifespan, enhance healthy aging, and mitigate aging‐related outcomes such as neoplasms and inflammation (Mao et al., 2018).

To comprehend the enigma of aging, it is imperative to investigate the downstream effectors of genes associated with aging. One such effector in InR signaling is the transcription factor FOXO, which undergoes phosphorylation by AKT and plays a crucial role in regulating the expression of various genes involved in stress resistance, metabolic regulation, cell cycle arrest, and lifespan extension (Kenyon, 2010; Martins et al., 2016). Furthermore, autophagy serves as an additional mediator implicated in the anti‐aging effects of InR signaling (Hansen et al., 2018; Nakamura & Yoshimori, 2018; Singh et al., 2019; Tóth et al., 2008). Inhibition of autophagy abolishes the lifespan extension effects observed in *C. elegans* with daf‐2 mutation (Uno & Nishida, 2016). Additionally, the mTOR signaling pathway, which can be activated by AKT and contributes to the regulation of autophagic flux and nutrition sense, is also encompassed (Johnson, 2018).

*Drosophila melanogaster* is a well‐established model organism extensively utilized in aging research, with several conserved signaling pathways associated with aging having been identified in this species. Notably, fly studies have contributed significantly to the discovery of key genes implicated in the aging process, including methuselah (mth) and I'm not dead yet (Indy) (Lin et al., 1998; Rogina et al., 2000). In contrast to mammals, fly insulin signaling pathway exhibits a simplified architecture characterized by a singular InR and a sole substrate named chico. This streamlined design facilitates targeted manipulation of insulin signaling activity (Giannakou & Partridge, 2007). Moreover, the utilization of fly genetics such as the UAS/Gal4 system further precise spatial and temporal regulation of insulin signaling activity (Brand & Perrimon, 1993; McGuire et al., 2004), thereby establishing flies as an exemplary model extensively employed in aging research (Piper & Partridge, 2018).

In addition to the ubiquitous intervention of the aging‐related genes, tissue‐specific manipulation of them also can elongate the lifespans of animals. For instance, knockout of InR in adipose tissue has been shown to prolong mouse longevity (Blüher et al., 2003), while inhibiting daf‐2 in neuron is also sufficient for lifespan extension (Roy et al., 2022). However, the downstream events following tissue‐specific intervention of insulin signaling remain unclear.

It is intriguing to uncover common aging signatures across diverse tissues through the manipulation of the same aging gene. Specifically, to determine whether inhibiting insulin signaling in different tissues can generate similar anti‐aging signatures and whether these signatures are consistent across various tissues, particularly at the proteomic level. Using a *Drosophila* model, we identified distinct proteomic changes in heads, midguts, and thoraxes during the aging process. Surprisingly, the knockdown of InR in fly CNS, muscles, and intestine resulted in lifespan extension. In particular, despite manipulating the same aging gene InR, there were also differential proteomic changes observed among these tissues. Moreover, knockdown InR in the intestine improved gut barrier integrity and enhanced anti‐ROS defense, leading to increased fly lifespan as well as resistance against bacterial invasion and ROS‐induced damage.

## 2 RESULTS

### 2.1 Proteomic analysis of aged *Drosophila* and corresponding tissues

**FIGURE 1:**
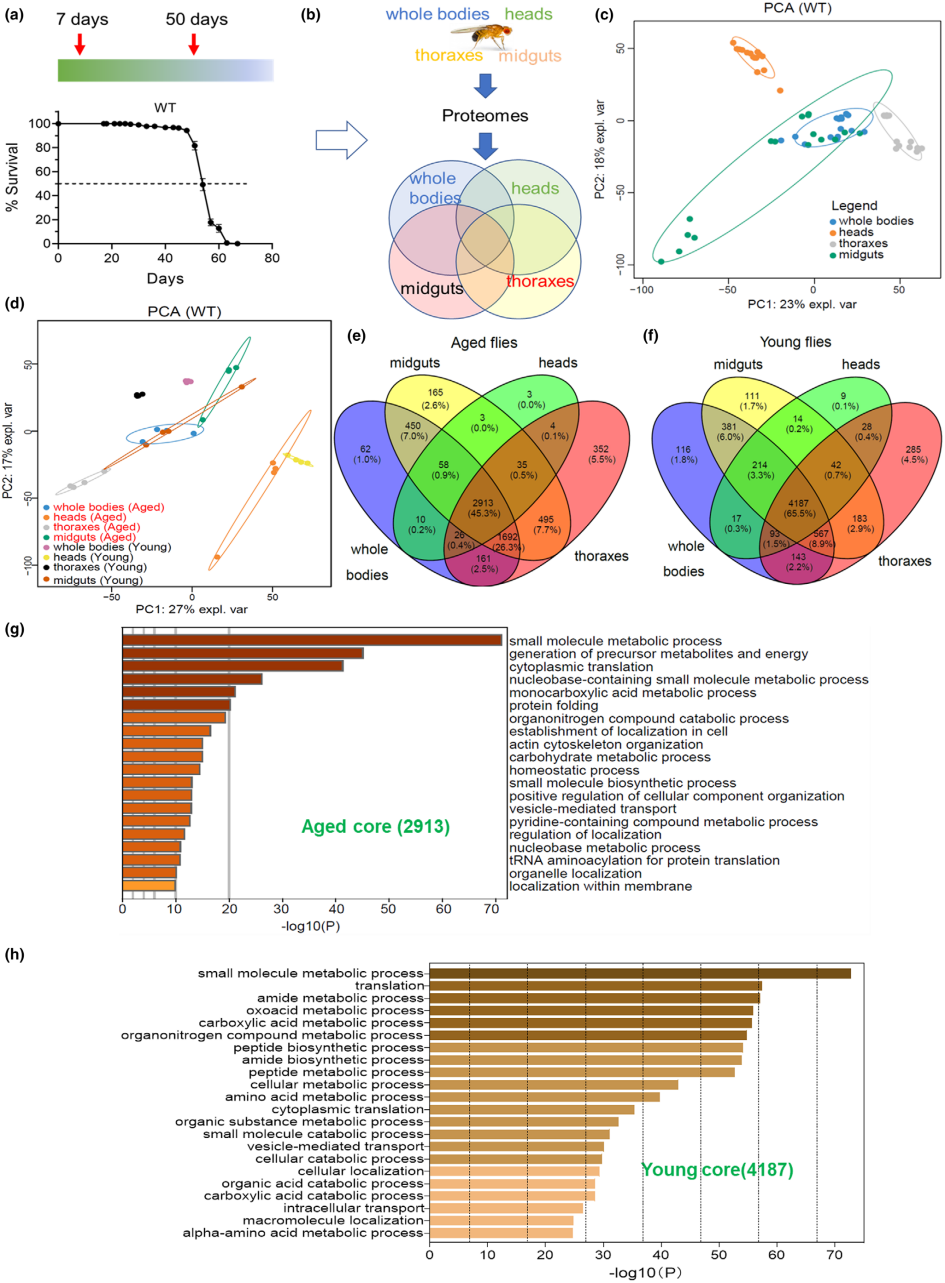
Proteomic analysis of aged *Drosophila* and corresponding tissues. (a) Lifespan of WT flies is recorded. Red arrows indicate the sampling time for collecting the fly samples for proteomic analysis (young flies, ~7 days old; aged flies, ~50 days old). (b) Sample collection and analysis schedules of proteomic study. Whole bodies, heads, midguts, and thoraxes are collected and analyzed by proteomes, to identify the common signature, the overlap strategy is used. (c, d) Principal Component Analysis (PCA) profiling of the proteomes results from separate samples. The biological replicates are indicated in the same color, the elliptic areas represent the standard error of the two components. (e, f) The Venn Diagram presents the core proteomes in aged flies (e) and young flies (f). (g, h) KEGG and GO functional enrichment analysis of the core proteomes of aged (g) and young flies (h).

The lifespan of wild‐type (WT) flies was initially assessed in accordance with standard conditions, revealing a median lifespan of ~52 days (Figure 1a). The proteomes of young and aged flies were analyzed by collecting ~7‐day‐old flies as the young group and ~ 50‐day‐old flies as the aged group (Figure 1a, indicated by arrows). Whole‐body samples, as well as fly heads, thoraxes, and midguts, were collected for quantitative proteomic analysis. The overlap strategy was employed to identify shared differences and tissue‐specific signatures in the proteomes that undergo alterations during the process of aging (Figure 1b). By utilizing principal component analysis (PCA), we successfully clustered the samples obtained from diverse tissues and whole bodies. The proteomes of midgut and whole‐body samples exhibited a higher degree of similarity (Figure 1c). Additionally, distinct alterations in proteomic profiles were observed in aged flies and tissues compared with their younger counterparts (Figure 1d), indicating age‐related changes in proteomes. Following quantification with DIA‐NN software, we successfully identified and quantified a total of 6538 proteins across all samples. Subsequently, a comparative analysis was performed on the proteins identified in these four sample types. The resulting Venn diagram revealed that 2913 proteins were common to all aged flies and tissues, while there were specific identifications of 3, 165, 62, and 352 proteins exclusively in the heads, midguts, whole bodies, and thoracic samples respectively (Figure 1e). The Venn diagram analysis of young fly samples revealed the identification of 4187 proteins that were commonly expressed in whole bodies, heads, midguts, and thoraxes. Additionally, tissue‐specific proteins were found to be present in numbers 116, 9, 111, and 285 across these respective samples (Figure 1f). The functional enrichment analysis of the 2913 common proteins identified in aged samples, as revealed by GO and KEGG enrichment analysis, demonstrated a significant enrichment primarily in small molecule metabolic processes, generation of precursor metabolites, energy metabolism, and cytoplasmic translation (Figure 1g). The 4187 common proteins identified in young flies were predominantly enriched in small molecule metabolic processes, translation, and amide metabolic processes (Figure 1h), suggesting the ubiquitous nature of these pathways across young tissues.

### 2.2 Identification of the specifically regulated proteins in aged flies and tissues

**FIGURE 2:**
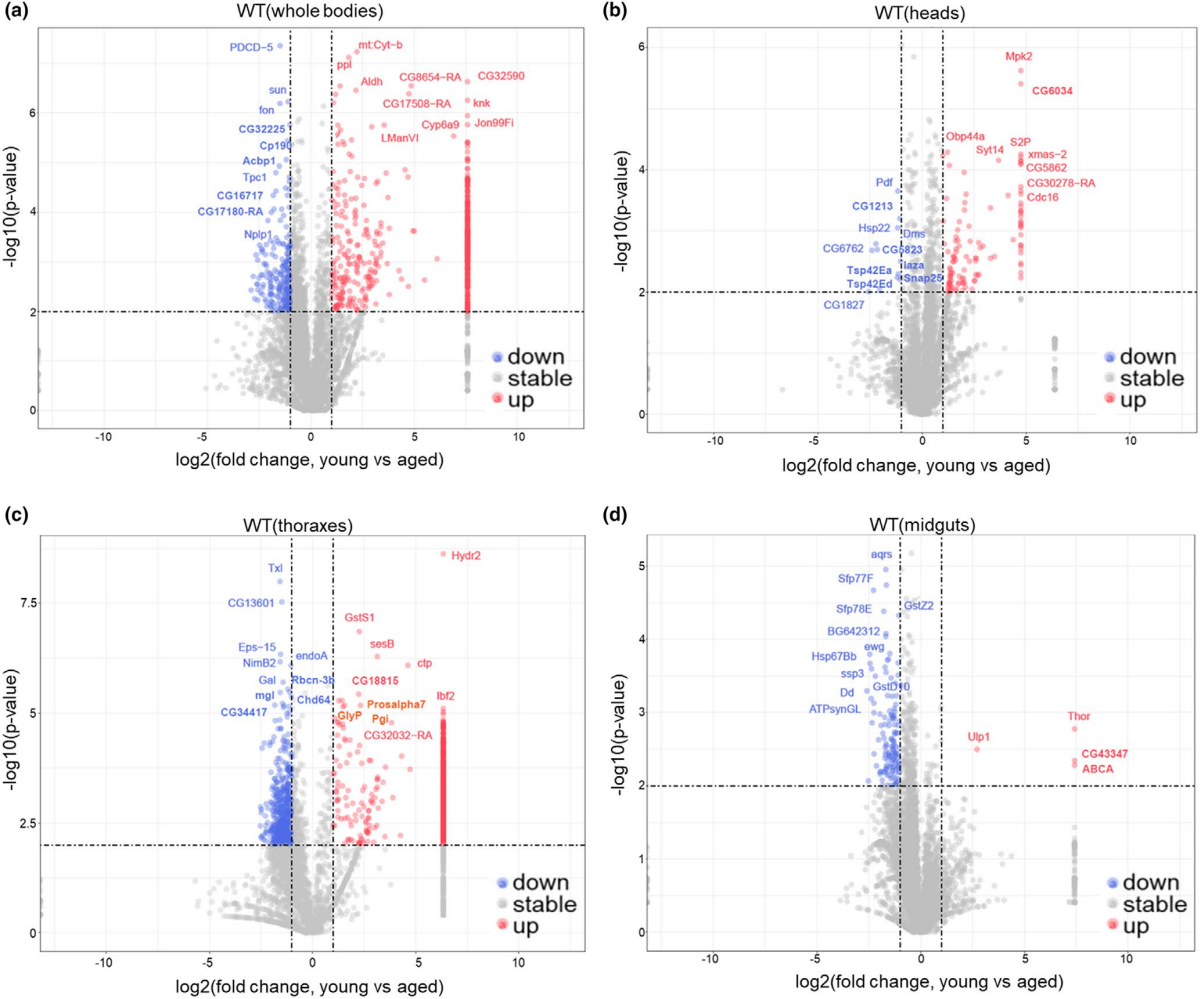
Identification of the specifically regulated proteins in aged flies and tissues. (a–d) Volcano plotting of the certain upregulated and downregulated proteins in aged (a) whole bodies, (b) heads, (c) thoraxes, and (d) midguts. −1og10(*p*‐value) is used as the cut‐off index.

To identify proteins that exhibit tissue‐specific upregulation or downregulation, we performed a differential analysis using a significance threshold of *p*‐value <0.01 and fold change >2, as depicted in the volcano plot figures (Figure 2a–d). Accordingly, the protein levels of PDCD‐5, sun, fon, CG32225, Cp190, Acbp1, Tpc1, CG167171, CG17180 and Nplp1 exhibited significant low levels in young flies (Figure 2a). Conversely, the protein levels of mt: Cytb, ppl, Aldh, CG8654, C G32590, CG17508, kink, LManVI, Cyp6a9, and Jon99Fi were markedly higher in young flies (Figure 2a). In aged heads, the protein levels of Pdf, CG1213, Hsp22, Dms (Drosomycin), CG6762, CG5823, laza, Tsp42Ea, Tsp42Ed, Snap25 and CG1827 were significantly upregulated. Conversely, Mpk2, CG6034, Obp44a, Syt14, S2P, xmas‐2, CG5862, CG30278, and Cdc16 exhibited downregulation in fly heads (Figure 2b). The protein levels of Txl, CG13601, Eps‐15, endoA, NimB2, Gal, Rbcn‐3b, mgl, Chd64, and CG34417 were found to be upregulated in the thoracic tissues. Conversely, Hydr2, GstS1, sesB, ctp, CG18815, Ibf2, Prosalpha7, GlyP, Pgl, and CG32032 exhibited downregulation (Figure 2c). In addition, the protein levels of aqrs, Sfp77F, GstZ2, Sfp78E, BG642312, ewg, Hsp67Bb, ssp3, GstD10, Dd, and ATPSynGL were found to be upregulated in midguts. Furthermore, the levels of Thor and CG43347 were downregulated in aged midguts (Figure 2d). Interestingly, we did not observe any consistent alterations in the expression of specific proteins across the aged whole body and typical tissues.

### 2.3 Analyzing the proteomes commonly regulated in aged flies and tissues

**FIGURE 3:**
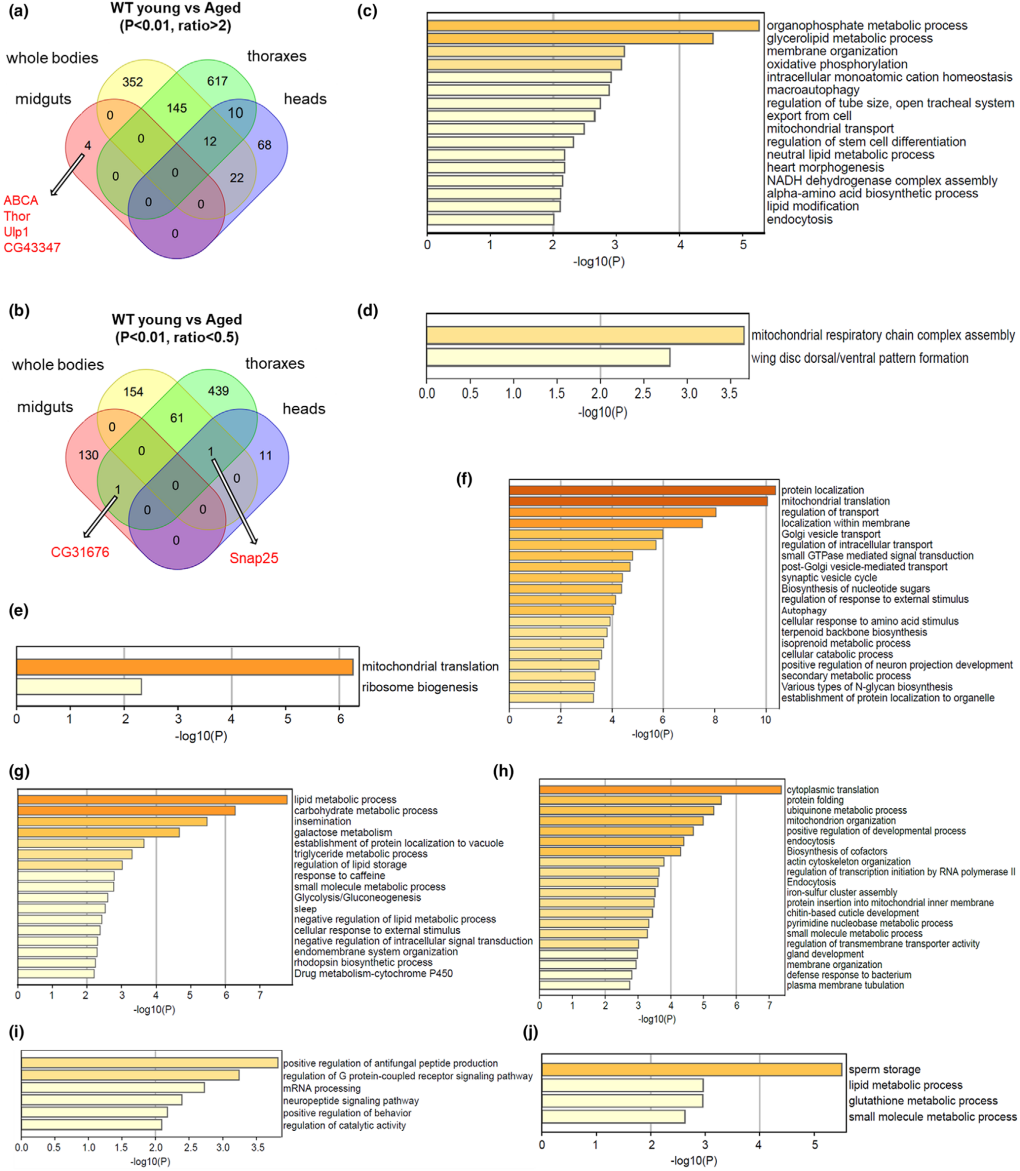
Analyzing the proteomes commonly regulated in aged flies and tissues. (a) Venn diagram plotting of the commonly and tissue specifically downregulated proteins across the aged flies and tissues. (b) Venn diagram plotting of the commonly and tissue specifically upregulated proteins across the aged flies and tissues. (c) KEGG and GO functional enrichment analysis of the 145 proteins which are commonly downregulated in thoraxes and whole bodies. (d) KEGG and GO functional enrichment analysis of the 61 proteins which are commonly upregulated in thoraxes and whole bodies. (e–g) KEGG and GO functional enrichment analysis of the proteins, which are specifically downregulated of aged fly tissues. (e) Specifically down‐regulated proteome of aged heads, (f) thoraxes, and (g) whole‐body samples. (h–j). KEGG and GO functional enrichment analysis of the proteins, which are specifically upregulated in aged fly tissues. (h) Specifically upregulated proteome of thoraxes, (i) whole‐body samples, and (j) midguts.

To further investigate the proteins commonly regulated across the whole body and tissues during the aging process, we performed a comprehensive analysis of the proteome from diverse tissue types and presented the findings using a Venn diagram (Figure 3a,b). Interestingly, no specific proteins exhibited common upregulation or downregulation patterns based on the Venn diagram analysis (Figure 3a,b). However, a total of 12 proteins exhibited common downregulation in whole body, thoracic, and head samples (Figure 3a and Table 1), while only one protein showed upregulation in aged whole bodies, thoraxes, and heads (Figure 3b).

**TABLE 1:** Commonly downregulated proteins across heads, thoraxes, and whole bodies.

List	Gene ID	Gene symbol	Description
Q9VCN6	42753	Cdc16	Cell division cycle 16
Q8MLT8	246523	CG30278	Uncharacterized protein
Q9VVK2	39974	CG6034	Uncharacterized protein
Q9VSF4	38917	Dhps	Deoxyhypusine synthase
M9MRN7	10178831	CG42846	Uncharacterized protein
P11996	33274	Lsp1beta	Larval serum protein 1 beta
P47947	35473	TpnC41C	Troponin C at 41C
Q24117	31405	ctp	Cut up
Q7KV26	32285	IP3K2	Inositol 1,4,5‐triphosphate kinase 2
Q9VQ62	33374	Npc2a	Niemann‐Pick type C‐2a
Q7K084	35789	Obp44a	Odorant‐binding protein 44a
Q9VM12	34022	CG5958	Uncharacterized protein

Among the 12 commonly downregulated proteins, there were five proteins with unknown function. The function‐characterized proteins included ctp, TpnC41C, Npc2a, Cdc16, Obp44a, and Lsp1beta (Table 1), which were respectively involved in regulating the axonal proteasome transport (Eastwood et al., 2021; Kreko‐Pierce & Eaton, 2017), and tubular muscle function (Chechenova et al., 2017), regulating the antibacterial response (Shi et al., 2012), controlling chromosome replication (Heichman & Roberts, 1998), regulating lipid metabolism (Yin et al., 2024), and metamorphosis and reproduction (Roberts et al., 1991).

The protein level of synaptosome‐associated protein 25 kDa (Snap25), a constituent of the SNARE complex involved in regulating vesicle exocytosis and calcium homeostasis, was found to be upregulated in fly heads, thoraxes, and whole‐body samples (Figure 3b), suggesting potential alterations in exocytosis dynamics and calcium balance within aged tissues (Antonucci et al., 2016; VanGuilder et al., 2010).

Notably, the proteomic changes observed in thoracic samples and whole‐body samples exhibited a higher degree of similarity. Specifically, 145 proteins were downregulated in both sample types, which were functionally enriched in processes such as organophosphate metabolism, glycerolipid metabolism, membrane organization, oxidative phosphorylation, intracellular monoatomic cation homeostasis, macroautophagy, regulation of tube size, and export from cell transport pathways. Additionally, these proteins were also involved in mitochondrial transport and regulation of stem cell differentiation as well as neutral lipid metabolic process, heart morphogenesis, NADH dehydrogenase complex assembly, alpha‐amino acid biosynthetic process, lipid modification, and endocytosis (Figure 3c). Further investigation of the proteins clustered in the organophosphate metabolic process revealed a predominant presence of proteins exhibiting oxidoreductase and transferase activities (Figure S1a).

Interestingly, the proteins that harbored the oxidoreductase activity were Gpdh1, Sgp, Adh, Sod2, Dhfr, and CG30427 (Figure S1b). In particular, Sod2 has been established as a key regulator of aging, as its decreased levels resulted in reduced fly lifespan, while overexpression of Sod2 extended yeast longevity (Fabrizio et al., 2004; Phillips et al., 1989). The downregulated transferases identified in this study were CG1227, CG7328, CG7718, and PKa‐C2 (Figure S1c). These transferases have demonstrated the ability to facilitate phosphor group transfers. Additionally, Pss, a phosphatidylserine synthase enzyme that impacts fly longevity, was also downregulated (Park et al., 2021).

The cluster of glycerolipid metabolic processes encompasses proteins with acyltransferase and fatty‐acyl‐CoA reductase activities, involved in glycerophospholipid biosynthesis and organelle membrane formation (Figure S1d). The downregulated acyltransferases were oys, Taz, mino Avc and fri, and the fatty‐acyl‐CoA reductases were wat, Sgp and CG30427 (Figure S1d). Additionally, within the group of commonly downregulated proteins, 10 proteins exhibited enrichment in the membrane organization cluster. These included oys, Rab7, Taz, scrambs, Vps60, CG17765, CG8111, EMC6, sesB, and CG10237; six of which were implicated in the endomembrane system (Figure S1e). Notably, Vps60 is known to participate in the formation of the ESCRT‐III complex for regulating endosome function by initiating alternative ESCRT‐III filaments (Pfitzner et al., 2023). Additionally, Rab7, a small GTPase involved in membrane trafficking and disease, regulates endosome/lysosome function (Zhang et al., 2009), and EMC6, an ER membrane protein that interacts with Rab5A, is involved in autophagy regulation (Li et al., 2013).

Functional network profiling using String DB also revealed a protein–protein interaction network comprising seven proteins that exhibited reduced expression in aged flies and were involved in the oxidative phosphorylation process (Figure S1f). Notably, five of these proteins were the subunits of mitochondrial respiratory complex I (mt: C‐I), namely ND1, 3, 5, 8, and B14.7 (Figure S1f), suggesting a decline in the functional capacity of mitochondrial complex I with age, which is consistent with previous studies on aging transcriptome (Zane et al., 2023).

Given the proposed role of macroautophagy in regulating aging through its involvement in cellular recycling (Nieto‐Torres & Hansen, 2021), we investigated the downregulated macroautophagy proteins commonly observed in thoracic and whole‐body samples (Figure 3c,f and Table 2). The downregulation of Vps13D, Trpml, and Rab3‐GAP (related to lysosome function), Emc6, and Atg4b (related to autophagy regulation) suggests the reduction in autophagy in these aged samples.

**TABLE 2:** Downregulated macroautophagy‐related proteins.

List	Gene ID	Gene symbol	Description
Q9VU08	39448	Vps13D	Vacuolar protein sorting 13D
Q9VW35	40152	Trpml	Transient receptor potential cation channel, mucolipin
Q9VF80	41868	Atg4b	Autophagy‐related 4b
Q9VKB9	34626	Rab3‐GAP	Rab3 GTPase activating protein
Q9VBZ8	42992	EMC6	ER membrane protein complex subunit 6

Moreover, a total of 61 proteins were consistently upregulated in both thoraxes and whole bodies, primarily enriched in the regulation of assembly for mitochondrial respiratory chain complex (RCC) as well as dorsal–ventral pattern formation in wing disks (Figure 3d). The upregulated proteins CG9065, CG42496, CG14757, Sirup, and CG12107 were found to be clustered in mitochondrial Rcc assembly (Figure S1g). Additionally, the proteins CG1943, gammaSnap1, and nuf were observed to cluster in wing disk dorsal–ventral pattern formation (Figure S1h). However, the relationship between the increased expression of these proteins and aging remains unclear.

Furthermore, a total of 22 proteins exhibited consistent downregulation in both whole body and head samples (Figure 3a and Table 3), including the lectin‐33A, bin3, Bet5, c(3)G, btv, Cog6, nAChRbeta3, Roc1a, Mekk1, Naa20A, Rbp6, Cpr65Av, Snx21, fdl, and Nepl18 (Table 3). The functional enrichment analysis revealed that the downregulated proteins in the heads and thoraxes were significantly enriched in regulating Golgi apparatus organization and protein metabolic processes (Figure S1i–S1k). Furthermore, the protein CG31676, which exhibits potassium ion transporter activity, exhibited upregulation in thorax and midgut samples (Figure 3b).

**TABLE 3:** Commonly downregulated proteins in heads and whole bodies.

MyList	Gene ID	Gene symbol	Description
Q7K480	35552	bin3	Bicoid‐interacting protein 3
Q9W5E1	31014	Roc1a	Regulator of cullins 1a
Q8MSQ4	42253	Mekk1	Mekk1
Q9VVE5	39919	Rbp6	RNA‐binding protein 6
Q9U3V9	44271	xmas	Xmas
Q9VKL8	53516	lectin‐33A	Lectin‐33A
M9PCM2	14462701	CG44000	Uncharacterized protein
A1ZAC2	36802	CG7786	Uncharacterized protein
Q9VA95	43590	Bet5	Blocked early in transport 5
Q9VF37	47765	c(3)G	Crossover suppressor on 3 of Gowen
Q0E8P6	35073	btv	Beethoven
M9PCI1	318843	CG43394	Uncharacterized protein
Q9V564	35953	Cog6	Component of oligomeric Golgi complex 6
Q9VPQ8	33228	nAChRbeta3	Nicotinic Acetylcholine Receptor beta3
Q9W087	38229	CG2021	Uncharacterized protein
M9PI14	32962	Naa20A	N(alpha)‐acetyltransferase 20 A
A4VCL2	42784	CG31145	Uncharacterized protein
Q8I0P8	318014	Cpr65Av	Cuticular protein 65Av
Q9VQG1	33466	Snx21	Sorting nexin 21
Q8WSF3	250735	fdl	Fused lobes
Q7K3M6	37275	CG8654	Uncharacterized protein
Q9VAS2	43410	Nepl18	Neprilysin‐like 18

The protein levels of 10 proteins were downregulated in both the thoracic and head tissues, including three proteins with unknown functions. Notably, Sps2, Aprt, mRpS31, mRpL53, gek, Trs31, and ben exhibited reduced protein levels in both the heads and thoraxes (Table 4). Among them, Trs31 was implicated in the regulation of autophagy (Zou et al., 2018), which is consistent with the observed decrease in autophagy‐related proteins both in thoraxes and whole bodies (Table 4). The reduction in mRpL53 and mRpS31 suggests a potential decline in mitochondrial translation during aging, which will be further discussed subsequently.

**TABLE 4:** Commonly downregulated proteins in aged heads and thoraxes.

List	Gene ID	Gene symbol	Description
P12426	48224	Aprt	Adenine phosphoribosyltransferase
P35128	32358	ben	Bendless
Q9W1B0	37858	gek	Genghis khan
A1Z9J6	3772367	mRpL53	Mitochondrial ribosomal protein L53
Q9VUX1	39749	mRpS31	Mitochondrial ribosomal protein S31
Q9VKY8	34397	Sps2	Selenophosphate synthetase 2
Q7K2Q8	36641	Trs31	TRAPP subunit 31
Q9VBH7	43170	CG14544	Uncharacterized protein
Q9VYV6	32121	CG2444	Uncharacterized protein
Q9VC87	42906	CG18528	Uncharacterized protein

### 2.4 Analyzing the proteomes specifically downregulated in aged flies and tissues

According to the results obtained from Venn diagram analysis, a total of 68 proteins exhibited specific downregulation in aged fly heads. Notably, these proteins were predominantly enriched in crucial biological processes such as mitochondrial translation and ribosome biogenesis (Figure 3e). The proteins involved in mitochondrial translation can be further classified into two groups: the components of the mitochondrial large ribosomal subunits, including mRpL18, 21, 35, and 36; and the components of the mitochondrial small ribosomal subunits, including mRps7 and 18A (Figure S2a). These proteins form a functional network that is enriched in mitochondrial translation processes, indicating a decline in mitochondrial translation in aged heads. Interestingly, a decrease in mitochondrial ribosomal proteins and mitochondrial complex I components has also been observed in thoraxes (Table 4, Figure S1f, and Figure 3c).

The thoracic samples exhibited a specific downregulation of 617 proteins (Figure 3a), which were predominantly enriched in the regulation of protein localization, mitochondrial translation, and transport and localization with the membrane (Figure 3f). The proteins functionally enriched in protein localization were further categorized into cellular macromolecule localization, vesicle‐mediated transport, and membrane‐associated localization (Figure S2b). The cellular macromolecule localization‐related proteins were predominantly enriched in GTPase activity, molecular carrier activity, signal sequence binding, and lipid transporter activity (Figure S2c). The proteins exhibiting GTPase activity were further categorized into ATP hydrolysis activity, including Kif3C, SERCA, ATP8A, and CG1598; and proteins are functionally associated with endosomes, Golgi apparatus, and lysosomes, including Rab3, 5, 6, 8, 14 and 18; Arl1 and 8; Arf79F; as well as ATP8A (Figure S2d).

Clustering of the Rab GTPase indicates a functional decline in endosomes during the process of aging. The downregulated proteins with molecular carrier activity, including Fs(2)Ket, Tnpo, emb, and Arts, were functionally grouped to nucleocytoplasmic carrier activity. Additionally, EMC4, EMC1, and EMC5 were functionally clustered as ER membrane complexes (Figure S2e), suggesting that the nucleocytoplasmic transport and ER transport are disrupted in aged thoraxes. Interestingly, further functional profiling of signal sequence binding proteins revealed that Nup58 was also clustered within the nucleocytoplasmic transport network (Figure S2f). The majority of proteins exhibiting lipid transporter activity were primarily involved in the transport of phospholipids (Figure S2g), encompassing subduced, cert, rdgBbeta, CG30392, ATP8A, and CG32485.

Moreover, the majority of proteins clustered in mitochondrial translation were identified as constituents of a functional network that encompassed both mitochondrial large ribosomal subunits and small ribosomal subunits comprising multiple mRpL and mRpS proteins (Figure S2h). Additionally, this network encompassed proteins involved in mitochondrial RNA metabolic processes (CG14231 and CG14450) as well as those exhibiting Aminoacyl‐tRNA ligase activity (LeuRS‐m, TyrRS‐m, and IleRS‐m) (Figure S2h). This finding is consistent with the observation indicating a decline in the mitochondrial translation network within aged heads (Figure S2a).

The proteins involved in the regulation of transport exhibited functional enrichment in GTP binding, protein‐containing complex binding, small GTPase binding, GTPase regulator activity, and actin filament binding (Figure S2i). String DB profiling further revealed the downregulation of AMPK‐alpha, which plays a crucial role in regulating longevity (Ulgherait et al., 2014). Additionally, a reduction in the protein responsible for mRNA export from the nucleus and regulation of actin filament dynamics was observed (Figure S2i). Notably, muscle contraction‐related proteins Mhc and Tm2 exhibited specific downregulation in the thoraxes.

Furthermore, a total of 352 proteins exhibited specific downregulation in whole‐body samples (Figure 3a), primarily associated with functional enrichment in lipid metabolism, carbohydrate metabolism, and insemination processes (Figure 3g). The proteins involved in lipid metabolic processes were predominantly clustered within the functional categories of lipase activity, phosphotransferase activity, and acyltransferase activity (Figure S2j). Among these proteins, CG11608, sl, CG11598, CG6962, CG31872, CG17097, CG18284, CG7910, and CG5162 were enriched in a functional network possessing lipase activity (Figure S2k). Additionally, CG6962, CG15743, Sl, and Ocrl also exhibited phosphoric ester hydrolase activity. The proteins CLS, CG6790, Gk2, Alg7, Ack, Cds, and Pis were found to cluster in phosphotransferase activity (Figure S2l,m). The proteins CG1941, FASN1, nes, CG16904, and CG9459 exhibited acyltransferase activity (Figure S2m). Notably, FASN1, CG16904, and CG9459 also demonstrated fatty acid synthase activity (Figure S2m), suggesting that lipid metabolism is disturbed in aging.

Proteins involved in the metabolic process of carbohydrates can be categorized into hydrolase activity (hydrolyzing O‐glycosyl compounds), carbohydrate binding, and racemase and epimerase activity (Figure S2n). The proteins involved in hydrolase activity clustering were predominantly associated with maltose alpha‐glucosidase and alpha‐mannosidase activities (Figure S2o,p), including MalA1, A3, and A8, as well as LManII, III, V, VI, and Mal‐A3. The proteins that were found to be enriched in insemination formed a functional network, which included sexual reproduction and mating behavior, consisting of 12 proteins (Figure S2q). This suggests that the decline in reproductive ability is caused by aging.

### 2.5 Analyzing the proteomes specifically upregulated in aged flies and tissues

According to the Venn diagram analysis, a total of 11 proteins were found to be specifically upregulated in fly heads (Figure 3b and Table 5). These proteins include well‐characterized functional molecules such as Myosuppressin (Ms), Hsp22, Tsp42Ea, Tsp42Ed, laza, and Pdf. Additionally, several function‐unknown proteins including CG1213, CG1827, CG6762, CG7497, and CG5823 were also identified among these upregulated proteins (Table 5).

**TABLE 5:** The proteins specifically upregulated in aged heads.

List	Gene ID	Gene symbol	Description
O96690	43193	Pdf	Pigment‐dispersing factor
Q9VNU2	40468	laza	Lazaro
P02515	3772576	Hsp22	Heat shock protein 22
P61849	44324	Ms	Myosuppressin
Q9VVJ1	39966	CG7497	Uncharacterized protein
Q7JWV7	35613	Tsp42Ed	Tetraspanin 42Ed
Q7K1I4	59178	Tsp42Ea	Tetraspanin 42Ea
Q8MR45	35989	CG1827	Uncharacterized protein
Q7JVN6	40728	CG1213	Uncharacterized protein
Q9VX10	32768	Srxn	Sulfiredoxin
Q9VEJ4	42105	CG5823	Uncharacterized protein

Among the function known proteins, Hsp22, a mitochondrial small heat shock protein in *Drosophila*, is known to be induced by stress and exhibits preferential expression during the aging process, it has been proposed as a potential biomarker for assessing fly aging and stress response (Morrow & Tanguay, 2015).

The levels of 439 proteins were specifically upregulated in thoracic tissues, exhibiting significant enrichment in cytoplasmic translation, protein folding, ubiquinone metabolic process, mitochondrion organization, positive regulation of the developmental process, endocytosis, and biosynthesis of cofactors (Figure 3b,h). The upregulated proteins associated with cytoplasmic translation primarily encompassed the large and small ribosomal subunits, including RpL6, 8, 10, 13, 16, 19, 29, 35a, 36a, and 37a; RpS19a, 21, 27, and 29; the translation initiation factors eIF3i and eIF2beta; as well as CNBP, and the components of ribonucleoprotein complexes such as Clbn (Figure S3a).

The proteins associated with protein folding were clustered within the functional networks of Chaperonin TCP1 and protein folding/refolding (Figure S3b). The Chaperonin TCP1 network comprises CCT2, 4, 6, and 8, which play a crucial role in facilitating the folding and assembly of evolutionarily conserved proteins (Kubota et al., 1995). The protein refolding network involved DnaJ‐60, −5, and ‐H, Hsc70‐5, Hsp27, and Droj2. Additionally, the protein folding network included jdp, Ero1L, P58IPK, shv, mlt, Pfdn2, Pfdn6, and Cdc37. The upregulation of the protein folding/refolding network represents a cellular response to the age‐related increase in protein misfolding stress (Wodrich et al., 2022).

The components involved in ubiquinone metabolism, including Coq3, 5, 7, 8, and 9, exhibited upregulation in the thoraxes (Figure S3c). Furthermore, proteins involved in mitochondrial organization exhibited upregulation in aged thoraxes, particularly certain subunits of mitochondrial complex I such as CG3262, CIA30, ND‐AGGG, and ND‐PDSW. Additionally, the levels of mitochondrial envelope proteins Tom70, Tim8, Tim10, and CG31229 (a core component of the TIM22 complex) were also increased in aged tissues (Figure S3d).

The whole‐body samples exhibited a specific upregulation of 154 proteins (Figure 3b), which were predominantly enriched in the positive regulation of antifungal peptide production, G‐protein coupled receptor signaling pathway regulation, and mRNA processing (Figure 3i), consisting with the previous transcriptome analysis (Zane et al., 2023). The upregulated Rel, spz, and grass were found to be significantly enriched in the Toll pathway (Figure S3e) (Valanne et al., 2011). The upregulated proteins involved in the G‐protein coupled receptor signaling pathway include sun, FOXO, CG42450, and Git (Figure S3f). Interestingly, both the sun and its receptor methuselah (mth) have been proposed as negative regulators of aging (Cvejic et al., 2004). Interestingly, the expression of the anti‐aging protein FOXO was found to be upregulated in the thoraxes (Figure S3f), potentially compensating for the decline in DNA‐binding activity associated with aging (Birnbaum et al., 2019). The protein levels of mRNA splicing factors, including U4‐U6‐60K, Prp38, Sf3b5, U2af38, Srrm234 (involved in alternative mRNA splicing), ecd (involved in U5 snRPN pre‐mRNA splicing complex), and CG2931 were found to be upregulated and formed a functional network (Figure S3g).

A total of 130 proteins were specifically upregulated in aged midguts, exhibiting further enrichment in sperm storage, lipid metabolic process, and glutathione metabolic process (Figure 3j). These proteins primarily clustered in the regulation of sperm storage (Figure S3h), lipase activity, and oxidoreductase activity (Figure S3i), as well as glutathione transferase activity (Figure S3j). The proteins exhibiting glutathione transferase activity were further organized into a functional network comprising glutathione transferases GstZ2, GstD10, GstE9, and GstE10 (Figure S3j).

The enrichment of seminal fluid proteins involved in sperm storage regulation may be attributed to the strong connection and cross‐talk between midguts and gonads (White & Wolfner, 2022). This includes the Serine protease Seminase (Sems) and aquarius (aqrs), which are implicated in regulating the proteolytic process, as well as the sex peptide (SP) that is involved in regulating midgut morphology and physiology (Figure S3h) (White et al., 2021). Collectively, these findings suggest an age‐associated alteration in the morphology and physiology of midguts. Furthermore, considering the potential involvement of Sems and aqrs as secreted proteases in the regulation of gut barrier integrity (Biancheri et al., 2013; Edogawa et al., 2020), we thus hypothesized that age‐related disturbances might occur in the midgut barrier function. To assess the barrier function of aged midguts, we employed the Smurf assay and 70KD‐Dextran permeability assay to evaluate intestinal permeability (Pandey et al., 2023; Rera et al., 2012). Interestingly, both food blue and 70KD‐Dextran‐FITC exhibited increased permeability in aged midguts (Figure S4a–e), suggesting a decline in the functionality of the intestinal barrier within aging.

### 2.6 Proteomic analysis of flies and tissues with InR RNAi

Given that insulin signaling has been established as a crucial pathway in aging, specifically the downregulation of InR and chico effectively extends the lifespan of flies (Clancy et al., 2001; Tatar et al., 2001). Our proteomic analysis results revealed the downregulation of *Thor* in aged midguts (Figure 2d), indicating an upregulation of insulin signaling. This finding was further validated through *Thor*, tobi qPCR, and AKT\p‐AKT immunoblot assays (Figure S5a–d). Therefore, we proceeded to suppress midgut insulin signaling by knocking down InR using the NP1‐Gal4 driver.

**FIGURE 4:**
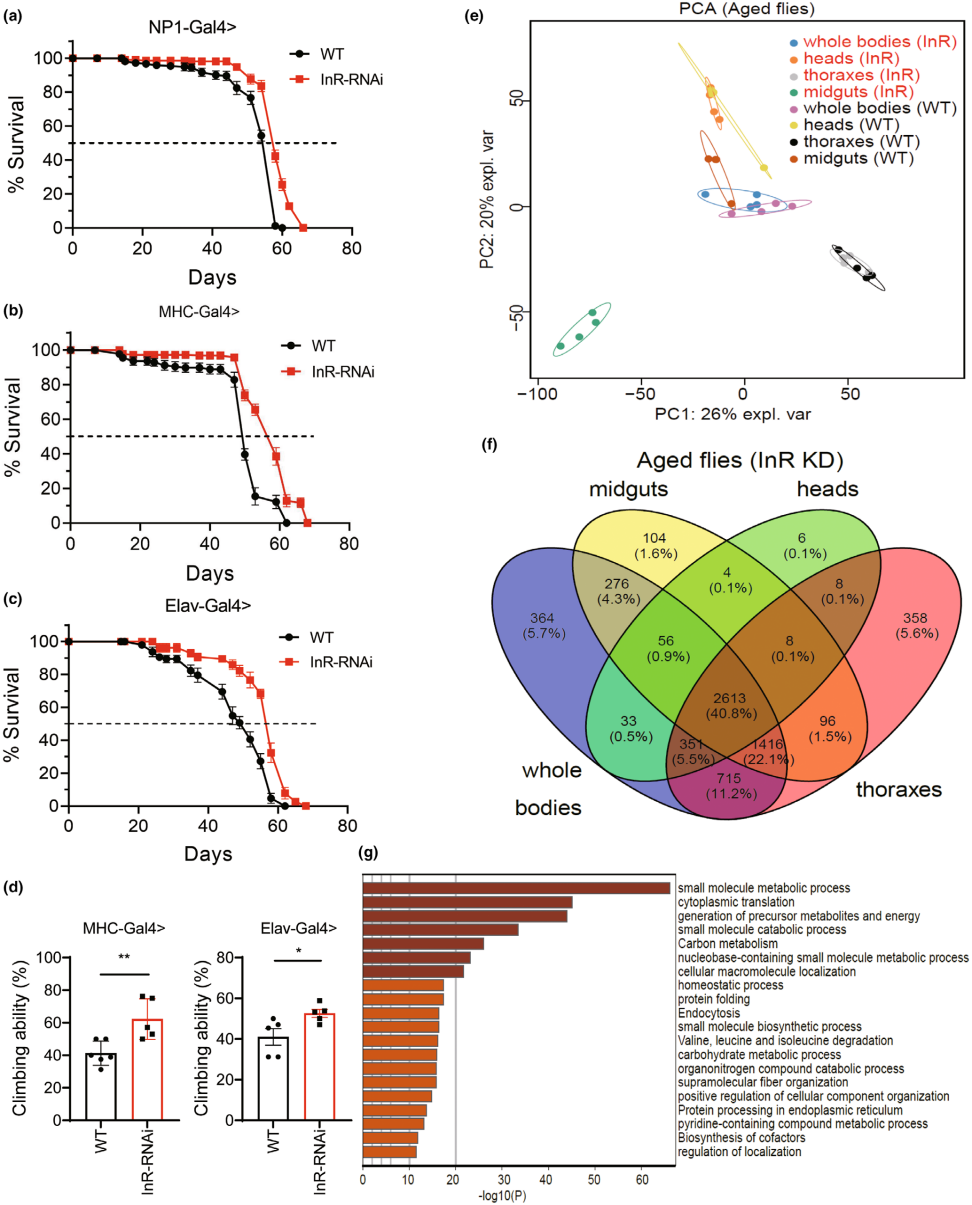
Proteomic analysis of flies and tissues with InR knockdown. (a–c) Lifespan recording of the WT flies and InR RNAi flies. (a) The lifespans for WT and midgut‐specific InR RNAi flies. NP1‐Gal4 is used to drive InR RNAi in fly midgut enterocytes. (b) The lifespans for WT and muscle‐specific InR RNAi flies. MHC‐Gal4 is used to drive InR RNAi in fly indirect flight muscles. (c) The lifespans for WT and neuron‐specific InR RNAi flies. Elav‐Gal4 is used to drive InR RNAi in the fly central nervous system (CNS). (d) The climbing ability of WT and InR RNAi flies. Left panel: InR is specifically knocked down in fly indirect flight muscle by using the MHC‐Gal4 driver. Right panel: InR is specifically knocked down in fly CNS by using the Elav‐Gal4 driver. *p < 0.05, **p < 0.01. (e) Principal component analysis (PCA) profiling of the proteomes results from separate samples. The biological replicates are indicated in the same color, the elliptic areas represent the standard error of the two components. (f) Venn diagram profiling of the quantified protein groups across the tissue, the core proteome including 2613 proteins is indicated. (g) KEGG and GO functional enrichment profiling of the core proteomes including 2613 proteins in (f), the top 20 enriched GO and KEGG terms are presented here.

As a result, NP1‐Gal4 mediated knocking down of InR in midgut enterocytes significantly elongated fly lifespan, the medium lifespan was extended from ~55 days to 60 days (Figure 4a and Figure S5e). Interestingly, the knockdown of InR in both the CNS and indirect flight muscle tissues also resulted in a similar extension of lifespan (Figure 4b,c, Figure S5e), with the medium lifespan extension from ~50 days to ~58 days respectively (Figure 4b,c). Further measurement of fly climbing ability confirmed the anti‐aging effects of InR RNAi. Specifically, knocking down InR in muscle and CNS significantly enhanced the climbing ability of aged flies(Figure 4d).

To investigate the common anti‐aging characteristics across diverse tissues with InR RNAi, we further examined proteomes of midguts, heads, and thoracic tissues. According to the PCA profiling, there was a pronounced disparity observed in the proteome of InR RNAi midguts compared with that of WT midgut and other tissues with InR RNAi (Figure 4e), indicating a substantial alteration in the proteomic composition of midguts upon knockdown of InR. A total of 2613 core proteins were consistently identified in both InR RNAi flies and tissues (Figure 4f), which were primarily enriched in small molecule metabolic processes, cytoplasmic translation, and generation of precursor metabolites and energy (Figure 4g). These findings further support the notion that these pathways remain consistent across different tissues and genetic manipulation.

### 2.7 Up‐ and downregulated proteins in InR RNAi flies and tissues

**FIGURE 5:**
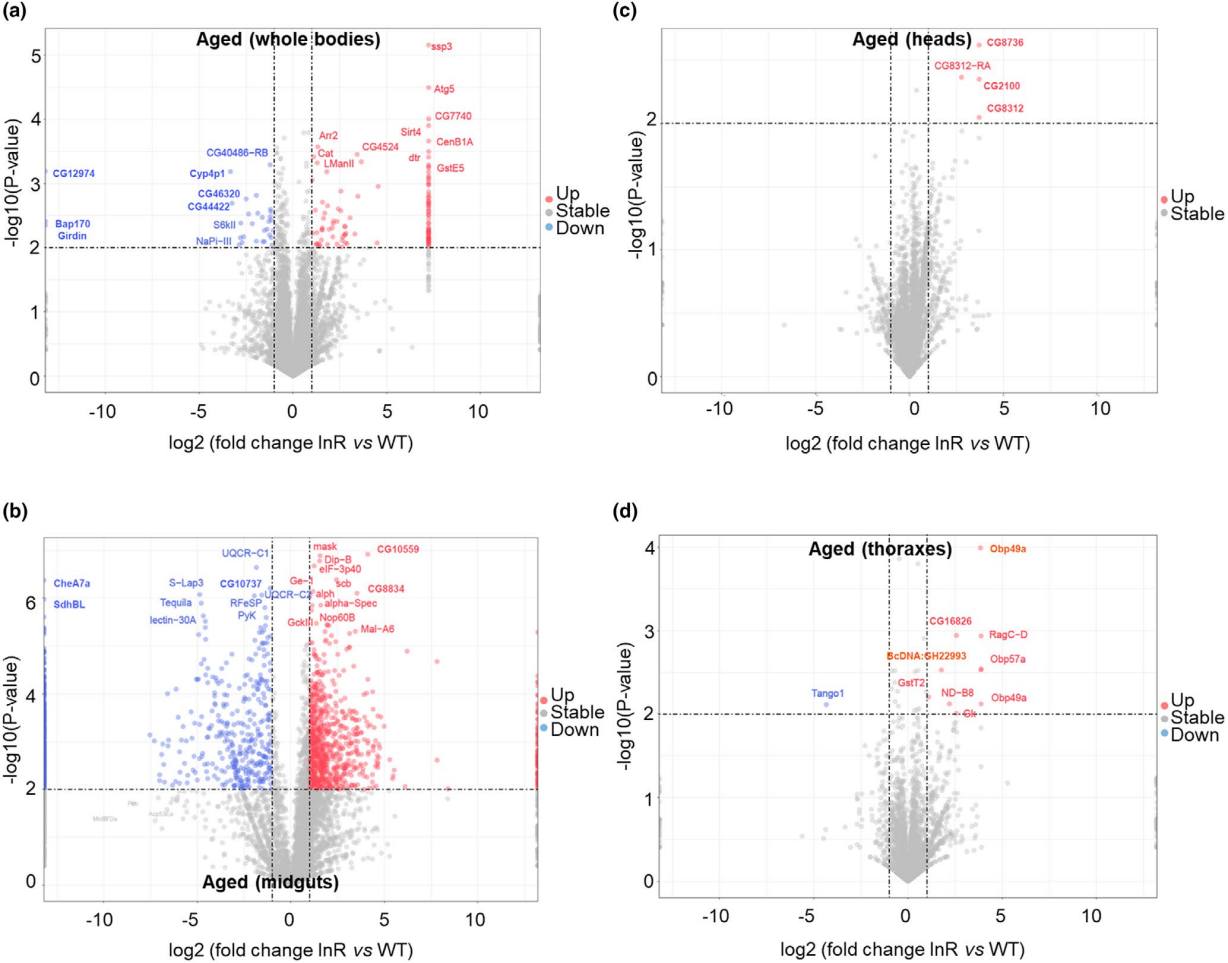
Specifically alerted proteins in InR RNAi flies and tissues. (a–d) Volcano plots the upregulated and downregulated proteins in InR RNAi flies and tissues, with the ‐log10 adjusted *p*‐values and log2 fold changes. (a) The changed proteins in whole‐body samples with InR RNAi, (b) the changed proteins in midguts samples with InR RNAi, (c) the changed proteins in heads samples with InR RNAi, and (d) in thoracic samples with InR RNAi. The upregulated proteins are presented in red color, the downregulated proteins are presented in blue color.

According to the volcano plotting, ubiquitious knockdown of InR resulted in the upregulation of ssp3, Atg5, CG7740, CenB1A, GstE5, Sirt4, dtr, CG4524, Arr2, Cat and LamnII, and the downregulation of CG40486, CG12974, Cyp4p1, CG46320, CG44422, S6KII, Bap170, Girdin, and Napi‐III (Figure 5a). Knockdown of InR in midgut enterocytes led to a decrease in the protein levels of UQCR, ChcA7a, S‐lap3, CG10737, SdhBL, Tequila, RFeSP, lectin‐30A, and Pyk. Conversely, it increased the levels of mask, CG10559, Dip‐6, Eif‐3P40, Ge‐1, scb, alph, CG8834, alpha‐Spec, Nop60B, GckIII and Mal‐A6 (Figure 5b). However, downregulating InR in fly CNS only increased the levels of CG8736, CG8312, and CG2100 (Figure 5c). Knockdown of InR in the indirect flight muscles resulted in decreased levels of Tango1 and increased levels of Obp49a, CG16826, RagC‐D, Obp57a, GstT2, ND‐B8, and Gk (Figure 5d).

We further employed an overlapped strategy to analyze the proteomes of young flies and InR RNAi flies, aiming to elucidate shared molecular signatures associated with anti‐aging effects. Interestingly, the protein levels of CG8736 were found to be significantly elevated in young individuals and InR RNAi heads (Figure S6a,b). Similarly, protein levels of ND‐B8, RagC‐D, CG6984, and Obp57a exhibited consistently high in both young and those InR RNAi thoracic tissues (Figure S6c,d). Notably, a total of 100 proteins were found to be downregulated in both young and InR RNAi midguts (Figure S6e,f), which were primarily enriched in sperm storage and alpha‐amino acid catabolic processes (Figure S6g,h). Interestingly, the upregulated proteases Sems and aqrs, as well as SP in aged midguts, exhibited reduction upon InR RNAi (Figure S6h), implying that InR RNAi may enhance the barrier function of midguts. Additionally, proteins involved in alpha‐amino acid metabolism included ppl, hgo, and GstZ2 (Figure S6h).

### 2.8 Proteomes commonly and tissue specifically regulated in InR RNAi flies and tissues

**FIGURE 6:**
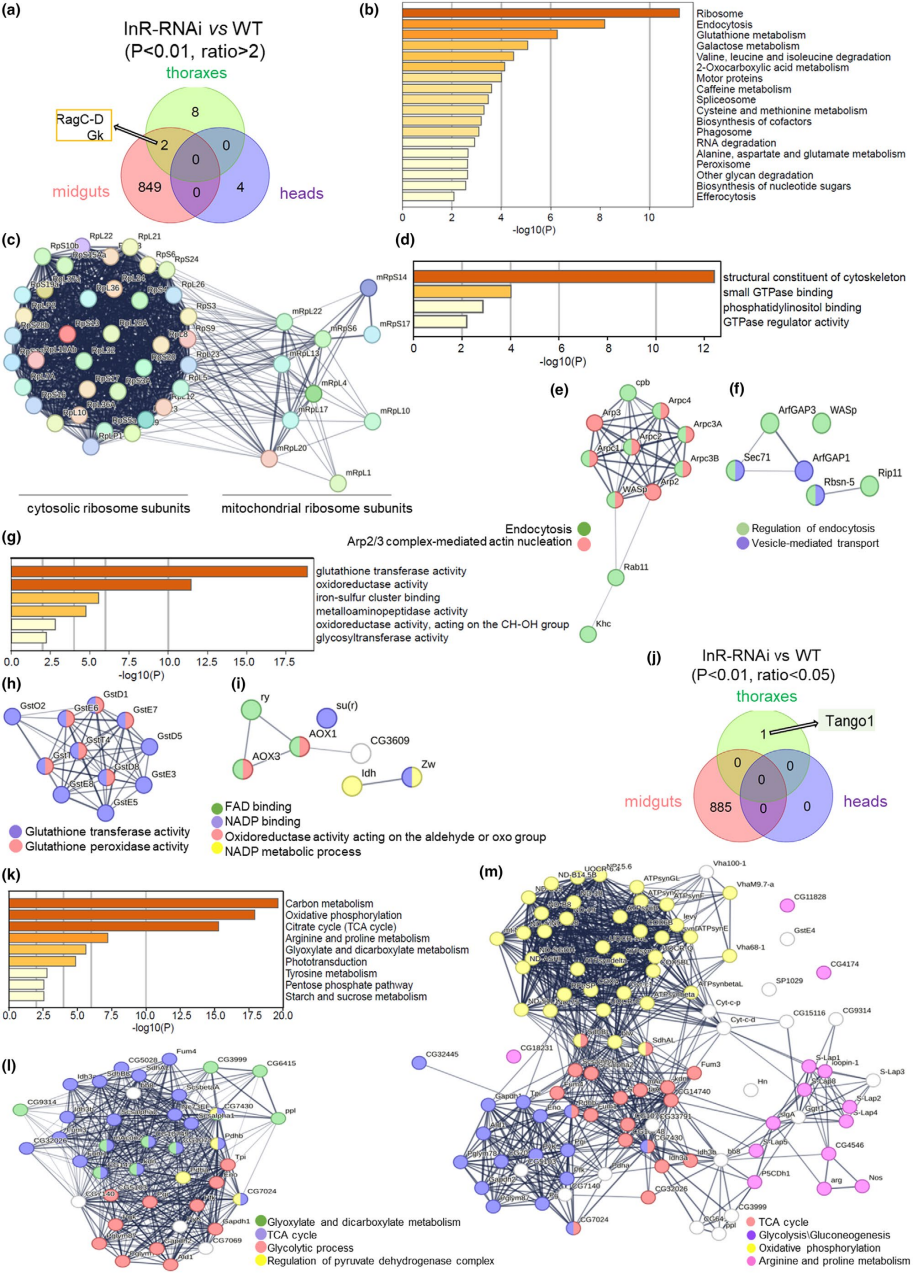
Proteins commonly and tissue specifically regulated in InR RNAi flies and tissues. (a) Overlap analysis of the proteins upregulated in thoraxes, midguts, and heads with InR RNAi. The 2 commonly upregulated proteins in the thoraxes and midguts are indicated, and the number of tissue specifically upregulated proteins are presented in the circle areas. (b) KEGG and GO functional enrichment analysis of the 849 proteins upregulated in midguts. The top 20 enriched items are presented here. (c) StringDB functional network plotting of the specifically upregulated midguts' proteome related to the ribosome in (b). (d) GO functional enrichment analysis of the molecular functions of the upregulated midgut proteins involved in endocytosis presented in (b). (e, f) StringDB functional network plotting of the specifically upregulated midguts' proteins involved in the structural constituent of the cytoskeleton (e) and small GTPase binding (f) presented in (d). (g) Go functional enrichment analysis of the molecular function of the upregulated proteins involved in glutathione metabolism presented in (b). (h, i) StringDB functional network plotting of the specifically upregulated proteins with the glutathione transferase activity (h) and oxidoreductase activity (i) presented in (g). (j) Overlap analysis of the proteins downregulated in thoraxes, midguts, and heads with InR RNAi. One specifically downregulated protein in the thoraxes is indicated, and the number of tissue specifically downregulated proteins is presented in the circle areas. (k) KEGG and GO functional enrichment analysis of the 885 proteins specifically downregulated in fly midguts with InR RNAi. The top 20 enriched items are presented here. (l, m) StringDB functional network plotting of the specifically downregulated proteins involved in carbon metabolism (l) and TCA cycle, Glycolysis\Gluconeogenesis process (Carbon metabolism), oxidative phosphorylation, and arginine, proline metabolism (m) presented in (k).

To further analyze the commonly upregulated and downregulated proteomes across InR RNAi tissues, we employed an overlapping strategy. Interestingly, no commonly upregulated proteins were identified across heads, midguts, and thoraxes; however, two proteins (RagC‐D and Gk) exhibited upregulation in both midguts and thoraxes (Figure 6a). RagC‐D is implicated in the regulation of lysosomal recruitment of activated mTOR (Ling et al., 2022; Liu & Sabatini, 2020), while Glycerol kinase (Gk) participates in acyl‐CoA generation and is involved in lipid and fatty acid metabolism (Morigny et al., 2021).

In particular, four proteins (CG8736, CG12384, CG2100, and CG8312) exhibited specific upregulation in the heads upon knockdown of InR in neurons (Figure 6a). Additionally, eight proteins (ND‐B8, Obp49a, CG16826, GstT2, CG31522, CG8768, CG6984, and Obp57a) were specifically elevated in the thoracic tissues with indirect flight muscle‐specific InR RNAi (Figure 6a and Table S1).

Furthermore, 849 proteins were specifically upregulated in the midguts with InR RNAi, exhibiting significant enrichment in the regulation of ribosome, endocytosis, and glutathione metabolism (Figure 6b). Intriguingly, the protein levels of cytosolic and mitochondrial ribosomal subunits were both upregulated in InR RNAi flies (Figure 6c), indicating an elevation and restoration in translation and protein synthesis. Consistently, the components of the spliceosome were also upregulated in InR RNAi midguts (Figure 6b).

**FIGURE 7:**
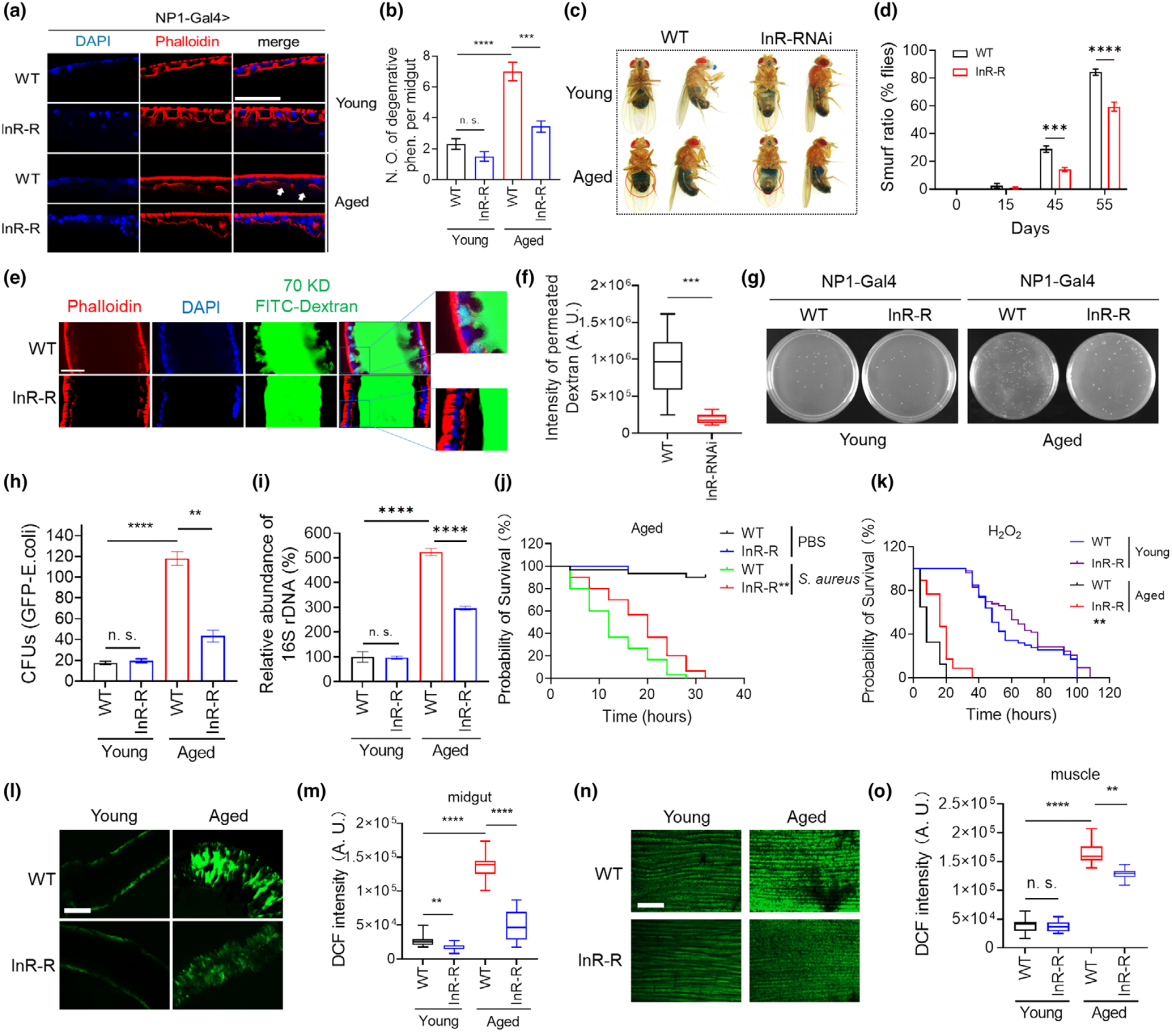
Silencing of InR specifically in the midgut delays the degeneration of the midgut barrier. (a) Aged midguts are stained with phalloidin‐TRITC to visualize the Actin network. NP1‐Gal4 is used to drive the knocking down of InR in midgut enterocytes. Scale bar = 50 um. (b) is the quantitative result of (a), ***p < 0.001, ****p < 0.0001. (c) Smurf assay is used to evaluate midgut permeability by using the food blue dye. The quantification of the proportion of flies exhibiting the Smurf phenotype during the aging process is presented in (d), ***p < 0.001, ****p < 0.0001. (e) Permeability of 70 kD FITC‐Dextran is observed in fly midguts. NP1‐Gal4 is used to drive the knocking down of InR in midgut enterocytes. Scale bar = 50 um. (f) is the quantitative result of (e), ***p < 0.001. (g, h) *E. coli* invasion in fly midguts is observed (g) and quantified (h). **p < 0.01, ****p < 0.0001. (i) The relative abundance of bacteria is assessed by quantifying the levels of 16 s rDNA using the quantitative PCR. ****p < 0.0001. (j) Survival curves reflect the sensitivity of WT and midgut‐specific InR RNAi flies to *Staphylococcus aureus* infection. **p < 0.01. (k) Survival curves reflect the sensitivity of WT and midgut‐specific InR RNAi flies to H_2_O_2_ treatment. **p < 0.01. NP1‐Gal4 is used to drive the knocking down of InR in fly midgut enterocytes. (l–o) ROS levels in fly midguts and muscles are evaluated by DCFH‐DA staining and (m, o) quantified. **p < 0.01, ****p < 0.0001. NP1‐Gal4 is used to drive InR RNAi in fly midgut enterocytes. Scale bar = 50um.

The upregulated proteins associated with endocytosis were predominantly clustered in the structural constituent of the cytoskeleton and small GTPase binding (Figure 6d–f). Interestingly, the majority of upregulated cytoskeletal proteins were found to be associated with the functional network of Arp2/3 complex‐mediated actin nucleation (Figure 6e) (Firat‐Karalar & Welch, 2011). This suggests that knocking down InR may lead to an enhancement of the Actin network in aged midguts through increasing actin nucleation, which will be further discussed in Figure 7. The upregulated proteins exhibiting small GTPase binding activity included Sec71, ArtGAP3, ArtGAP1, Rbsn‐5, Rip11, and WASp (Figure 6f), which were primarily implicated in the regulation of endocytosis.

The proteins involved in glutathione metabolism were further categorized into glutathione transferase and oxidoreductase (Figure 6g). The proteins involved in glutathione transferase activity formed a functional network that included several glutathione transferases and peroxidases, which played a role in combating oxidative stress (Figure 6h). This suggests an enhancement of antioxidant ability in the midguts with InR RNAi. The upregulated oxidoreductase activity was attributed to proteins binding to FAD, such as AOX1, AOX3, and ry, as well as proteins binding to NADP, like su(r) and Zw (Figure 6i).

According to the overlap analysis, we did not identify any commonly downregulated proteins across tissues. However, it was observed that only Tango1 protein exhibited specific downregulation in the thoraxes (Figure 6j). Tango1 is known to be involved in cargo loading and secretion processes, such as procollagen transport (McCaughey et al., 2021; Saito et al., 2009).

Intriguingly, the downregulation of InR in midguts resulted in a specific reduction in 885 proteins, which exhibited significant enrichment in carbon metabolism, oxidative phosphorylation, and the TCA cycle (Figure 6k–m **)**. The downregulated proteins in the midguts were specifically associated with a diminished functional network encompassing TCA cycles, glycolysis/gluconeogenesis, oxidative phosphorylation, and arginine and proline metabolism (Figure 6m). This suggests that the metabolic rates were reduced in the midguts of InR RNAi flies, potentially contributing to the observed lifespan‐extending effects resulting from InR RNAi (Miquel et al., 1982).

In conclusion, the knockdown of InR in fly midguts resulted in enhanced translation, improved actin network and antioxidant defenses, and reduced serine proteases, thereby potentially enhancing the barrier function of midguts.

### 2.9 Rejuvenating the midgut's barrier function through knockdown of InR

To further evaluate the barrier function of InR RNAi midguts, we first assessed the integrity of the actin network through Phalloidin‐TRITC staining, given that InR RNAi resulted in elevated protein levels of the actin nucleation components (Figure 6e). The degeneration of the Actin network in midguts was significantly decelerated by InR RNAi (Figure 7a,b). Consistently, the aged flies exhibited disturbed integrity of the midgut actin network (Figure S7a,b).

The Smurf assay further demonstrated that the knockdown of InR effectively enhanced the barrier functions of midgut cells (Figure 7c,d and Figure S7c) (Tatge et al., 2023). Moreover, the permeability of 70KD FITC‐dextran was also reduced in InR RNAi midguts (Figure 7e,f, Figure S7d,e), providing additional evidence for the enhancement of fly midgut barrier function. The barrier function was further validated by evaluating the permeability of bacteria. InR RNAi significantly reduced the midgut's permeability to GFP‐*Escherichia coli* (Figure 7g,h), and the amount of bacteria that permeated through (Figure 7i), resulting in increased resistance of flies to *Staphylococcus aureus* infection, as evidenced by an extended medium survival time from ~12 h to ~16 h (Figure 7j and Figure S7f).

Given that InR RNAi in midguts also enhances the functional network of glutathione metabolism, which contributes to antioxidant defense, it is plausible to suggest an improvement in the antioxidative capacity due to InR RNAi (Figure 6g,h). Interestingly, knockdown of InR in midgut enterocytes resulted in enhanced resistance of flies to H_2_O_2_ treatment, as evidenced by a significant increase in the medium survival time from ~8 to ~17 h (Figure 7k). The levels of reactive oxygen species (ROS) were significantly decreased in the midguts upon InR RNAi treatment (Figure 7l,m). Interestingly, the knockdown of InR specifically in the midguts also resulted in a reduction in ROS levels in fly muscles, indicating that midgut RNAi targeting InR can effectively inhibit the permeation of ROS species (Figure 7n,o).

Given the association between increased inflammation and age‐related decline of the gut barrier (Salazar et al., 2023), we investigated the expression of fly peptidoglycan recognition proteins (PGRPs) (Royet et al., 2011). We observed a significant upregulation in the expression of PGRP‐LC and PGRP‐SD in aged flies, while the knockdown of InR resulted in their reduction (Figure S8a,b).

Intriguingly, targeted downregulation of InR in midgut tissue resulted in a reduction in intestinal stem cell proliferation in aged midguts **(**Figure S8c,d), which is well‐documented as an age‐related change (Salazar et al., 2023). This further suggests that InR RNAi delays gut cell aging. Considering the association between mitochondrial dysfunction and aging progression (Salazar et al., 2023), we subsequently analyzed the mitochondrial membrane potential using tetramethylrhodamine ethyl ester (TMRE) staining (Rottenberg, 2023; Zorova et al., 2018). A significant decrease in TMRE signals was observed in aged midguts; interestingly, knockdown of InR partially restored these signals (Figure S8e,f), indicating a delay in the decline of mitochondrial membrane potential and suggesting that InR knockdown can slow down midgut cell aging and aging‐related functional decline. Interestingly, the overall levels of ubiquitination and ref(2)P (fly p62) were significantly diminished upon knockdown of InR in gut epithelial cells (Figure S8g,h), indicating a restoration of proteostasis. Furthermore, knockdown of InR specifically in midgut epithelial cells also improved climbing ability among aged flies (Figure S8i, flies aged for ~50 days), suggesting that preserving gut barrier function can effectively decelerate systemic aging.

### 2.10 The aging process in mice is accompanied by the degeneration of the duodenum barrier

**FIGURE 8:**
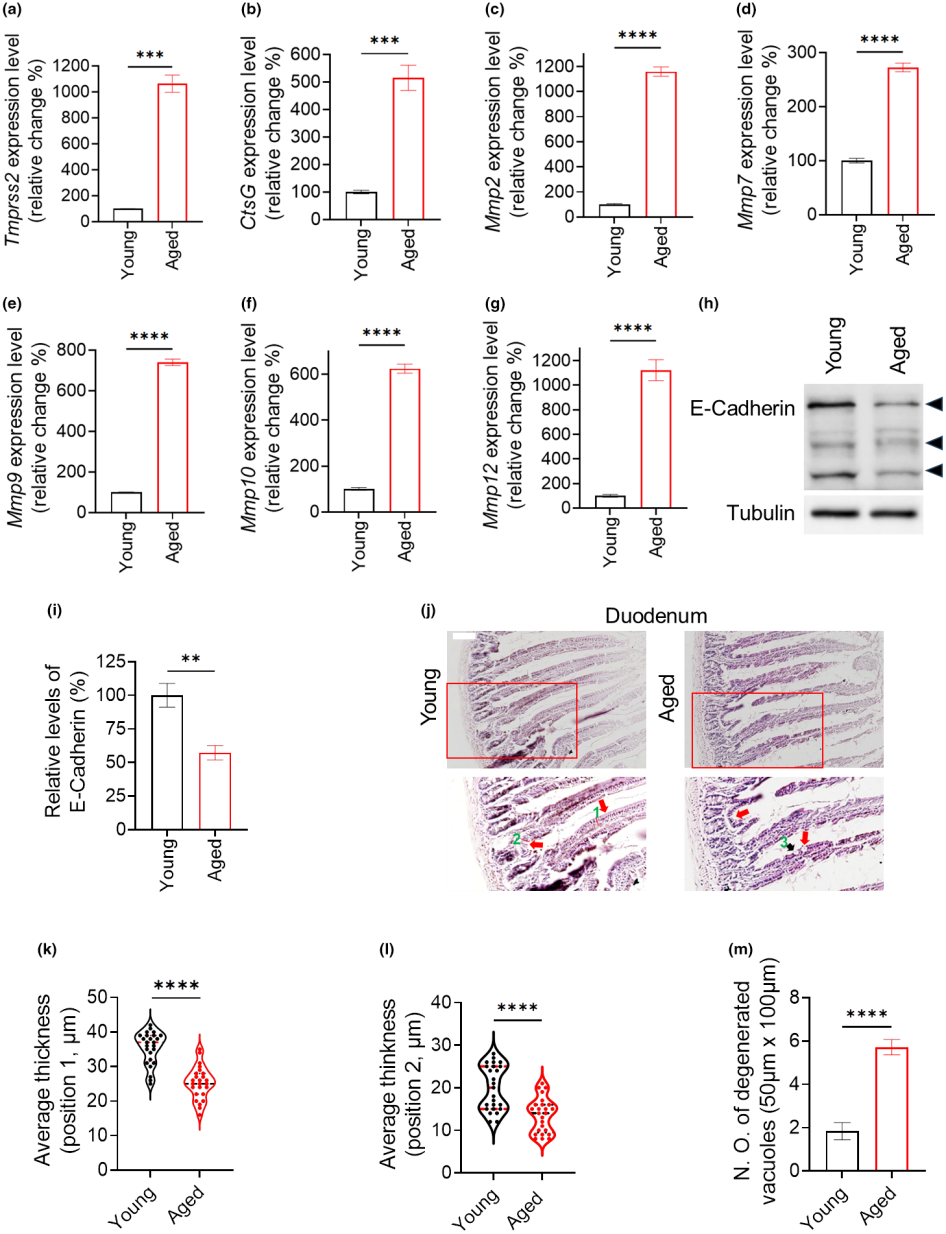
Mouse aging is associated with the degeneration of duodenum barrier. (a, b) The expression levels of serine proteases Tmprrs2 and CtsG in the mouse duodenum are quantified using quantitative PCR (qPCR). ***p < 0.001. (c–g) The expression levels of MMPs in mouse duodenum are analyzed by qPCR. ****p < 0.0001. (h) The protein levels of duodenum E‐cadherin are assessed via immunoblotting. Tubulin serves as the loading control. Arrowheads indicate the prominent cadherin bands. (i) is the quantitative result of (h), **p < 0.01. (j–m) Degeneration of duodenal epithelial barrier is assessed by histological sections and H&E staining. Scale bar = 50um. (k) and (l) are the quantitative results of the thickness at position 1 and 2 epithelial barriers respectively. (m) is the quantitative result of the degenerative vacuoles in the duodenum epithelial barrier. ****p < 0.0001.

To further validate our findings from the fly model, we investigated the expression of age‐associated serine proteases. Specifically, we observed an increase in the expression of transmembrane protease, serine 2 (TMPRSS2) (Figure 8a), which belongs to the type 2 transmembrane serine protease family and participates in extracellular matrix degradation and remodeling (Mukai et al., 2015; Schuler et al., 2021). Additionally, we found an increase in Cathepsin G (CtsG) expression (Figure 8b), a secreted serine protease involved in extracellular matrix and plasma protein degradation (Korkmaz et al., 2008). These results suggest that aged mouse duodenum also exhibits increased expression of extracellular matrix remodeling‐related serine proteases.

Furthermore, we analyzed several matrix metalloproteinases (MMPs) as they are known to be involved in intestinal epithelial barrier remodeling (Lee & Kim, 2022). Interestingly, MMP2, MMP7, MMP9, MMP10, and MMP12 were upregulated in aged duodenum samples (Figure 8c–g). This further supports our hypothesis that the integrity of the duodenal epithelial barrier may be influenced during aging.

Consequently, a comprehensive analysis was conducted to assess the integrity of the mouse epithelial barrier (Salazar et al., 2023). There was a notable downregulation in the protein levels of E‐cadherin, a key component involved in modulating adherent junctions (Daulagala et al., 2019), within the aged duodenum (Figure 8h,i). Furthermore, histological examination and hematoxylin and eosin (H&E) staining of duodenal sections revealed significant shrinkage and degeneration in the aged animals' epithelial barrier, characterized by thinning and increased presence of degenerative vacuoles (Figure 8j–m). These findings provide compelling evidence that age‐related reduction in epithelial barrier function is also observed in mouse duodenum, further supporting its conservation across species.

## 3 DISCUSSION

According to the proteomic analysis, distinct protein changes were observed across different tissues. However, further functional network analysis revealed some common signatures during aging, such as the decline of mitochondrial translation, glutathione metabolism, and autophagy. This suggests that different tissues experience similar functional disturbances rather than sharing the same altered protein species during aging. Therefore, it is currently challenging to identify a specific protein indicating ubiquitous aging across different tissues. Instead, highlighting a certain age‐related functional network becomes crucial in indicating systemic aging across tissues, particularly within networks associated with functional integrity. Previous studies have also examined the transcriptional signature of aged whole‐body samples. Although the Smurf phenotype is strongly associated with advanced aging and mortality, both Smurf flies and non‐Smurf flies exhibit similar regulation of their transcriptional signatures during aging, which share similarities with the aging transcriptome (Zane et al., 2023). Interestingly, when comparing our proteomic data with the analysis of whole‐body transcriptomics, several common changes can be observed. Specifically, increased inflammatory responses involving both Toll and immune deficiency (Imd) signaling pathways (Figure 3i and Figure S3e), are highlighted in both analyses (Zane et al., 2023). Furthermore, elevated Rel expression is detected in both transcriptomic and proteomic studies, further confirming increased inflammation in aged flies. Notably, enhanced stress responses such as protein folding/unfolding responses identified in the aging transcriptome can also be validated in our thoracic proteome (Figure S3b). Additionally, decreased levels of ribosomal proteins, OXPHOS activity, components of ETC, and fatty acid metabolism previously described in the transcriptomic analysis are further supported by our proteomic analysis (Figure 3c,e,g) (Zane et al., 2023), suggesting that proteomic and transcriptomic analyses reveal similar signatures associated with aging. Therefore, additional multi‐omics analyses could be considered to explore potential signatures related to aging by combining information from age‐related proteomes, transcriptomes, metabolomes, and post‐translational modifications.

Notably, the severity of the Smurf phenotype is more pronounced in several studies, particularly regarding entire body Smurfness (Zane et al., 2023). However, complete manifestation of entire body Smurfness also is not consistently observed in some studies (Bitner et al., 2020; Cai et al., 2021; Keshav et al., 2022), which may be attributed to variations in blue dye concentration (Keshav et al., 2022), differences in food composition (Cai et al., 2021), and variation of the Smurf phenotype (Bitner et al., 2020). Therefore, quantifying blue dye levels in aged flies may provide a more precise assessment.

Although InR RNAi can extend the lifespan of flies, there are distinct proteomic differences between young flies and those with InR RNAi. Compared with aged flies, a common signature has emerged‐lower levels of secreted serine proteases in the midguts of young flies and InR RNAi flies. Given that secreted serine proteases have been implicated in age‐related damage to the gut barrier (Edogawa et al., 2020), we propose that the barrier function of midguts is more robust in young flies and those with InR RNAi. Indeed, several assays assessing midgut barrier function have validated our hypothesis by demonstrating enhanced barrier function upon downregulation of InR (Figure 7).

Interestingly, the barrier function of midguts is disrupted during aging, leading to the invasion of pathogens and harmful materials. Knockdown of InR can enhance barrier function by increasing translation, improving actin network functionality, and enhancing antioxidant defense mechanisms. This is accompanied by a reduction in protease activity that may contribute to age‐related damage of the midgut barrier. Therefore, downregulation of InR leads to enhanced defense against pathogens and ROS‐induced damage. While previous studies have discussed the link between gut barrier function and aging (Branca et al., 2019; Kühn et al., 2020), our work provides novel insights into how manipulation of the aging gene InR can rejuvenate gut barrier function and contribute to increased longevity. These findings further emphasize the importance of maintaining healthy gut physiology as an effective strategy for slowing down systemic aging.

Serine proteases are involved in regulating gut barrier dynamics as previously described (Edogawa et al., 2020), since they are involved in regulating the levels of junction proteins and the activation of the proteinase‐activated receptors (Van Spaendonk et al., 2017), which can directly influence the intestinal permeability. Although the molecular mechanism is unclear, we also find that some serine proteases are upregulated in the aged midgut, which may help to explain the dysfunction of the fly midgut barrier. Accordingly, aging‐related upregulation of serine proteases also has been characterized in mammals. However, whether these serine proteases can directly regulate the intestinal barrier and their concrete contribution to systematic aging, are remained to be clarified. In addition, whether the serine proteases are involved in regulating other barriers, also needs to be investigated further. In addition to serine proteases, the change and role of other types of proteases in aging process, are worth to be clarified. It is worth noting that MMPs are also upregulated in aged mouse intestine, which are suggested to be included in regulating the intracellular matrix degradation and the integrity of the intestinal barrier (Van Spaendonk et al., 2017). Interestingly, characterizing the inhibitors of the proteases is also worth to be considered as an effective approach (Edogawa et al., 2020; Van Spaendonk et al., 2017), which can maintain the barrier's integrity and slow down systematic aging.

The change in ribosome proteins is differently regulated in young flies and InR RNAi flies. Particularlly, the levels of ribosomal proteins are increased in aged flies and further increased in InR RNAi flies, suggesting a different regulatory mechanism. Indeed, the reduction in protein turnover and translation is associated with longevity elongation (Swovick et al., 2021), which is consistent with our observation of the increment of cytosolic ribosomal proteins and translational factors. However, the increased cytosolic ribosomal proteins and mitochondrial ribosomal proteins in InR RNAi midguts may indicate a reprogram of protein turnover and mitochondrial function, thereby contributing to the enhancement of functional integrity against aging. Although sharing many similarities, the gut barrier in flies is a comparatively simple system compared with mammals (Salazar et al., 2023). However, both fly and mouse aging exhibit degeneration of epithelial barriers (Figures 7 and 8), which aligns with the observation that age‐related dysfunction of the intestinal barrier is prevalent across various organisms (Salazar et al., 2023). Consequently, impaired intestinal barrier function can lead to detrimental consequences, particularly increased permeability to harmful substances and bacteria, further compromising systemic functional integrity, and promoting cell death through mechanisms such as elevated redox species levels and inflammation. Therefore, preserving the functional integrity of the intestinal barrier is crucial for delaying systematic decline in healthy physiology and function. By targeting the knockdown of the aging factor InR in *Drosophila* (Piper & Partridge, 2018), we provide additional evidence supporting the critical role of intestinal integrity in systemic aging. Furthermore, pharmacological interventions or dietary modifications like the caloric restriction that reduce insulin signaling pathways (Klement & Fink, 2016), can be considered further.

## 4 MATERIALS AND METHODS

### 4.1 Fly strains and cross

The *Drosophila* stocks utilized in this study were mainly obtained from the Bloomington *Drosophila* Stock Center (BDSC). Wild‐type flies, UAS‐InR RNAi flies, Elav‐Gal4 flies, and MHC‐Gal4 flies were obtained from BDSC, while NP1‐Gal4 flies were obtained from the *Drosophila* Genetic Resource Center (DGRC). The Elav‐Gal4 flies were crossed with WT (y v; attP2, y+) and UAS‐InR RNAi flies to generate Elav‐Gal4/Y and Elav‐Gal4/Y; UAS‐InR RNAi/+ flies, respectively. Similarly, the MHC‐Gal4 and NP1‐Gal4 flies were crossed with WT and UAS‐InR RNAi flies to produce +/Y; MHC‐Gal4/+, +/Y; MHC‐Gal4/UAS‐InR RNAi, +/Y; NP1‐Gal4/+, and +/Y; NP1‐Gal4/+; UAS‐InR‐RNAi/+ flies. Flies were raised on standard corn meal under standard conditions.

### 4.2 Lifespan and climbing ability assay

The longevity and climbing ability assays were conducted according to previously described methods (Huang et al., 2019). Briefly, newly hatched male flies were collected and divided into at least 10 groups, flies were transferred to the fresh food every 2 days, and the number of dead flies was recorded every time and used to calculate the survival rate. For each treatment, a total of 200 flies were used. For the climbing ability assay, at least five groups of 15 aged flies each were used (~40 days old), and the ratio of flies capable of climbing a distance of 3 cm within an 8‐second time frame was calculated as the index of climbing ability. To evaluate the impact of midgut InR RNAi on the climbing ability of aged flies, a 50‐day aging period was implemented before conducting the assay.

### 4.3 Protein extraction and LC–MS/MS sample preparation

The flies' samples were collected from whole male fly bodies, heads, thoraxes, and midguts, with young flies aged for approximately 7 days and aged flies aged for approximately 50 days. These samples were used for protein extraction and referred to as “young” and “aged” samples, respectively. The dissected tissues and bodies of the flies were homogenized directly in lysis buffer (8 M urea, 1% protease inhibitors, Sigma‐Aldrich) on ice, followed by centrifugation at 12,000 rpm at 4°C. The protein concentrations were determined using the BCA kit (Thermo Fisher Scientific). Each replicate consisted of 20 μg of urea lysate, which was initially reduced with 10 mM DTT (Sigma‐Aldrich) for 60 min. This was followed by alkylation with 55 mM IAA (Sigma‐Aldrich) for 30 min in the absence of light before proceeding to digestion.

The proteins were subsequently subjected to overnight trypsin (Promega) digestion at 37°C, employing an enzyme‐to‐protein ratio of 1:100. The digestion process of urea samples was terminated by the addition of TFA (Sigma‐Aldrich) to achieve a final concentration of 0.1% (*v*/*v*). The peptides were desalted using C18 StageTips with SDB‐RPS resin (3 M EMPORETM), and the eluates containing the purified peptides were subsequently dried using a Speedvac system (Thermo Fisher Scientific). Following this, the peptides were reconstituted in 20 μL of 0.1% formic acid (FA) (Sigma‐Aldrich), and subsequently, 2 μL of protein solution was injected into the mass spectrometer (Thermo Fisher Scientific).

### 4.4 LC–MS/MS measurements

Peptides were loaded onto 30 cm reversed‐phase columns with an inner diameter of 75 μm, packed in‐house with C18 1.7 μm resin. The column temperature was meticulously maintained at 50°C using a specialized column oven to ensure optimal conditions. The Vanquish Neo UHPLC system (Thermo Fisher Scientific) was seamlessly and directly coupled online to the state‐of‐the‐art mass spectrometer, the Orbitrap Exploris 480 (Thermo Fisher Scientific). The peptides were subjected to a meticulous separation process utilizing an advanced binary buffer system, which entailed the utilization of buffer A (0.1% formic acid) and buffer B (100% acetonitrile plus 0.1% FA), both at a precise flow rate of 350 nL/min (for 60 min) during the DIA scan. The DIA methods were meticulously designed, incorporating a comprehensive MS1 scan (*m*/*z* = 300–1250) with an AGC target of 3 × 10^6^ and a maximum injection time of 60 ms (*R* = 60,000). Additionally, the DIA scans were carefully acquired at *R* = 30,000 using an AGC target of 3 × 10^6^ and an “auto” setting for injection time.

### 4.5 Raw data processing

The DIA‐NN software, version 1.8.1 (https://github.com/vdemichev/DIANN), was employed for handling MS raw files obtained through DIA mode. In library‐free mode, DIA‐NN searches were conducted using a protein sequence database comprising 13,821 entries from UniProt (UP000000803, *Drosophila melanogaster*). DIA‐NN allowed for a maximum of one missed cleavage and permitted up to two variable modifications per peptide, such as acetylation at protein N‐termini and methionine oxidation. The carbamidomethylation of cysteines was considered a consistent modification throughout the study. “Match between runs” (MBR) was implemented as necessary. In the absence of a library, we employed deep learning‐based spectra and retention time (RT) prediction. The generation of the library involved the implementation of both experiment‐wide and run‐specific precursor FDR filters established at a significance level of 1%.

The mass spectrometry proteomics data have been deposited to the ProteomeXchange Consortium (https://proteomecentral.proteomexchange.org) via the iProX partner repository with the dataset identifier PXD048120.

### 4.6 Bioinformatics data analysis

For DIA‐NN outputs, precursor intensities were aggregated into peptides using the MaxLFQ algorithm, which was implemented in the DIA‐NN R package (https://github.com/vdemichev/DIA‐NN‐rpackage/). Before conducting differential expression analysis, the protein intensities were normalized using median sample scaling. Significance testing of intensities was performed using a student *t* test, with *p*‐values <0.01 considered statistically significant. The KEGG and GO functional enrichment analysis was conducted using the MetaScape database (https://metascape.org/) (Zhou et al., 2019). Protein–protein interaction (PPI) enrichment analysis was performed utilizing the STRING database (STRING DB: https://cn.string‐db.org/) (Szklarczyk et al., 2023).

### 4.7 Quantitative PCR (qPCR) analysis and western blot

Approximately 10 whole male fly bodies and 20 fly tissues were collected for RNA extraction, following the manufacturer's instructions of TRIzol Reagent (Thermo Fisher Scientific). The cDNA synthesis was performed using the Transcript One‐Step gDNA Removal and cDNA Synthesis SuperMix kit (TransGen Biotech), followed by Real‐time PCR analysis with corresponding primers (Table S2), using the qPCR reaction mix (ChamQ Universal SYBR qPCR Master Mix). Real‐time PCR was performed and analyzed by QuantStudio 3 (Thermo Fisher Life technologies). Thirty midguts were prepared from both young and aged flies, homogenized in cell lysis buffer (Absin) containing phosphate inhibitor (Absin) and protease inhibitor (Absin), and mixed with 5 X SDS‐Loading buffer (TransGen Biotech). Protein samples were separated using a 12% SDS‐PAGE gel and subsequently transferred onto PVDF membranes (Merck). The PVDF membranes were probed with anti‐AKT (Cell Signaling Technology) and anti‐p‐AKT (Cell Signaling Technology) antibodies at a dilution of 1:2000, followed by incubation with HRP‐conjugated anti‐Rabbit antibody (Cell Signaling Technology) at a dilution of 1:2000. Finally, the membranes were analyzed using Super‐Sig ECL Chemiluminescent Reagent (Shenhua Biotech) and a Chemiluminescent Imaging System (Sagecreation Technology).

### 4.8 Smurf assay and phalloidin staining

The standard medium was prepared as described, with the addition of 2% food blue dye (SUGARMAN). Male flies were transferred onto the dyed medium and subsequently observed for extraintestinal leakage of blue dye, which was referred to as the “Smurf assay” (Rera et al., 2012). The proportion of Smurf flies relative to the total number of flies was calculated as the Smurf ratio. To quantify the food blue dye, Smurf flies were subjected to a 6‐h fasting to empty their gut contents. Subsequently, whole male flies were homogenized in 1xPBS and centrifuged at 12,000 rpm and 4°C. The resulting supernatants were collected and analyzed for absorbance at a wavelength of 629 nm.

To evaluate the permeability of midguts, a filter paper containing a solution of 70 kD Dextran‐FITC (Sigma‐Aldrich) at a concentration of 0.15 mg/mL was placed on the fly medium (Pandey et al., 2023). After a period of starvation for 12 h, ~40‐day‐old flies were transferred onto the Dextran and Phalloidin‐supplemented food and cultured for an additional 15–20 h. Subsequently, the midguts were collected and fixed in 4% paraformaldehyde (Sangon Biotech) before being stained with DAPI (Beyotime Biotech). The images were captured using a Zeiss LSM900 confocal microscope (Zeiss) and analyzed using ZEN Blue software (v3.7) from Zeiss.

### 4.9 ROS sensitivity and DCFH‐DA staining

To assess the sensitivity of flies to ROS damage, tert‐butyl hydroperoxide (5 μL/mL) (Sangon Biotech) was added to the standard fly food. Aged male flies were then transferred onto the hydroperoxide‐supplemented food, and their mortality was recorded at 4‐h intervals. The midguts and muscles of the flies were dissected and incubated with the DCFH‐DA probe (1:2000) (Beyotime Biotech) at 25°C for 30 min. Subsequently, they were washed three times with 1 × PBS (Sangon Biotech) and observed using a Zeiss LSM900 confocal microscope (Zeiss). Image analysis was performed using ZEN Blue software (v3.7, Zeiss). At least 10 independent images were captured for each treatment and were analyzed.

### 4.10 Bacteria invasion assay

To assess the sensitivity of male flies to invasion by *Staphylococcus aureus* (a gift from Prof. Cong Yi, Zhejiang University), a bacterial solution (O. D. = 2) was applied onto filter paper and plated on a standard fly medium. Young and aged flies were initially starved on filter paper soaked in dH_2_O for 12 h before being transferred to food containing bacteria. The number of deceased flies was recorded at 4‐h intervals, and the survival rate was subsequently calculated.

The *E. coli* invasion assay was performed similarly. A solution of *E. coli* (a gift from Prof. Xiaofeng Xia, Fujian Agriculture and Forestry University) with kanamycin resistance (O. D. = 2) was applied onto filter paper. Flies were reared on this medium for 12 h, followed by transferring to filter paper soaked in dH_2_O only for a 12‐h starvation period to deplete food and bacteria from their digestive tracts. Subsequently, fly homogenate was prepared using 1xPBS (Sangon Biotech) and plated on kanamycin plates (Sangon Biotech), which were then incubated at 37°C for an additional 18 h. The number of bacterial colonies was counted and quantified.

### 4.11 Tetramethylrhodamine ethyl ester perchlorate (TMRE) staining and immunofluorescence

The adult midguts were dissected in 1xPBS and subsequently incubated with 1 × TMRE detection solution (Beyotime Biotech) at room temperature for 30 min. Following this, the midguts were washed twice with the detection buffer for 5 min each and observed using a Zeiss LSM900 confocal microscope (Zeiss). At least 10 independent images were captured and analyzed per treatment.

For the immunofluorescence analysis of the midgut, male fly tissues were dissected in 1xPBS and fixed in 4% paraformaldehyde (Absin) for 30 min. Subsequently, they were washed three times with 1xPBS. The tissues were then permeabilized with 1xPBS containing 2‰ Triton X‐100 (Sangon Biotech) for 30 min and blocked with a solution of 10% goat serum (diluted in 1xPBS containing 1‰ Tween20, Sangon Biotech) for 1 h. Following this step, the samples were incubated at room temperature for 2 h with primary antibody against phosphor‐Histone3 (PH3) (Abcam). After washing three times with PBST (1xPBS containing 1‰ Tween20), the samples were further incubated with Alex594‐conjugated anti‐rabbit secondary antibody (Cell Signaling Technology) for 2 h. Following another round of washing three times with 1xPBST (containing 1‰ Tween20), midgut samples were incubated with DAPI for 10 min and subsequently washed again using 1xPBS. Finally, the samples were observed using a Zeiss LSM900 confocal microscope (Zeiss). Image analysis was performed using ZEN Blue software (v3.7, Zeiss). At least 10 independent images were captured and analyzed per treatment.

### 4.12 Mouse duodenum samples and experiments

The duodenum samples from both aged and young C57BJ mice were obtained from Cyagen Biosciences Inc. (China). Subsequently, the samples were cut and washed with 1xPBS before RNA extraction was performed according to the aforementioned protocol. Real‐time PCR analysis was conducted to determine the expression levels of mouse genes using the primers listed in Table S2. Additionally, protein extraction was carried out on the washed duodenum samples for immunoblots analysis using anti‐E‐cadherin antibody (Abcam) and anti‐Tubulin antibody (Abcam), following similar procedures as described above.

The duodenum from both aged and young mice was collected and fixed in 4% formaldehyde, followed by dehydration in anhydrous ethanol. Subsequently, the samples were immersed in methyl benzoate for 2 h and stepwise incubated at 60°C in a 1:1 mixture of methyl benzoate and melted paraffin before embedding with paraffin. Continuous paraffin sections were prepared using Leica HistoCore BIOCUT (German) and subjected to stepwise staining with Hematoxylin and Eosin (Beyotime Biotechnology).

### 4.13 Statistical analysis

Statistical analyses were performed using GraphPad Prism (v9.0), and significance was determined by conducting a *t* test analysis for two groups. The data are presented as the mean ± S. E. M.

## AUTHOR CONTRIBUTIONS

Y.P.H. conceived the manuscript and revised the manuscript. Y.P.H., C.Y.Z., J.L.W., T.Z.Y., and J.X.H. analyzed the data and wrote the manuscript. J.R.W. offered valuable suggestions. Other authors provided some experimental help. All authors approved the article.

## CONFLICT OF INTEREST STATEMENT

The authors declare no conflict of interest.

## Supporting information


**Data S1:** Supporting Information.

## Data Availability

The corresponding author can provide access to the original data upon request.
