# Supplementary material for: Proteomic analysis across aged tissues reveals distinct signatures and the crucial involvement of midgut barrier function in the regulation of aging
Source: Aging Cell. 2024 Sep 25;24(1):e14344. doi: 10.1111/acel.14344 (PMC11709110; doi:10.1111/acel.14344)

**SUPPLEMENTARY INFORMATION**

**Supplementary Tables**

**Supplementary Table 1. Up-regulated proteins in fly heads with InR RNAi**

| **List** | **Gene ID** | **Gene Symbol** | **Description** |
| --- | --- | --- | --- |
| Q9VR00 | 50178 | ND-B8 | NADH dehydrogenase (ubiquinone) B8 subunit |
| Q7JVM1 | 36399 | Obp49a | Odorant-binding protein 49a |
| Q9VK00 | 34741 | CG16826 | uncharacterized protein |
| A1Z7X7 | 246386 | GstT2 | Glutathione S transferase T2 |
| A0A0B4KF65 | 40567 | CG31522 | uncharacterized protein |
| Q8T0N5 | 36400 | CG8768 | uncharacterized protein |
| Q7K1C3 | 36926 | CG6984 | uncharacterized protein |
| Q8MKK0 | 246670 | Obp57a | Odorant-binding protein 57a |

| **Supplementary Table 2. qPCR primers** | | |
| --- | --- | --- |
| **Genes** | **Forward** | **Reverse** |
| *PGRP-LC* | AGGCCGTCACAGTTACAGTG | GTGGTGGCCAGTACGATACC |
| *PGRP-SD* | GACAGCATGGAAACTCCCTTG | GTTTTGCAGATTTTGCATGTGC |
| *tobi* | GTGCGACTTAGGAGAGGTGAT | GCTGTCAAAGTTGTCGAAGGT |
| *Thor* | CAGATGCCCGAGGTGTACTC | TCATGAAAGCCCGCTCGTAG |
| *InR* | AAGCGTGGGAAAATTAAGATGGA | GGCTGTCAACTGCTTCTACTG |
| *16S rDNA* | AGAGTTTGATCCTGGCTCAG | CTGCTGCCTYCCGTA |
| *rp49* | GCACCAAGCACTTCATCC | CGATCTCGCCGCAGTAAA |
| *CtsG* | CCCTACATGGCATTTCTTCTGAT | GTTAGGACGAAGTCTTCTCGC |
| *Mmp7* | CTTACCTCGGATCGTAGTGGA | CCCCAACTAACCCTCTTGAAGT |
| *Mmp9* | GCAGAGGCATACTTGTACCG | TGATGTTATGATGGTCCCACTTG |
| *Mmp10* | GAGCCACTAGCCATCCTGG | CTGAGCAAGATCCATGCTTGG |
| *Tmprss2* | TATGAGAACCACGGGTATCAGT | CGTTGTAATCCTCGGAGCATACT |
| *Mmp2* | ACCTGAACACTTTCTATGGCTG | CTTCCGCATGGTCTCGATG |
| *Mmp12* | GGGCTGCTCCCATGAATGAC | CCAGAGTTGAGTTGTCCAGTTG |
| *Actin-B* | GTGACGTTGACATCCGTAAAGA | GCCGGACTCATCGTACTCC |

**Supplementary Figures and Legends**

**Supplementary Figure 1. Analyzing the proteomes commonly regulated in aged flies and tissues**

**(A)**. KEGG and GO functional enrichment analysis of the commonly down-regulated organophosphate metabolic process-related proteins in thoraxes and whole bodies. **(B, C)**. StringDB functional network plotting of the proteins with the oxidoreductase activity and transferase activity in **(A)**. **(D)** StringDB functional network plotting of the proteins involved in glycerolipid metabolic process in main Figure 3B. **(E)**. proteins involved in membrane organization in Figure 3B. **(F)**. proteins involved in oxidative phosphorylation in Figure 3B. **(G)**. proteins involved in mitochondrial respiratory chain complex assembly and **(H)** wing disc dorsal/ventral pattern formation in Figure **3D**. **(I)**. KEGG and GO functional enrichment of 22 proteins that commonly down-regulated in heads and thoraxes. **(J, K).** Proteins involved in the Golgi apparatus and regulation of protein metabolic process in (**I**).


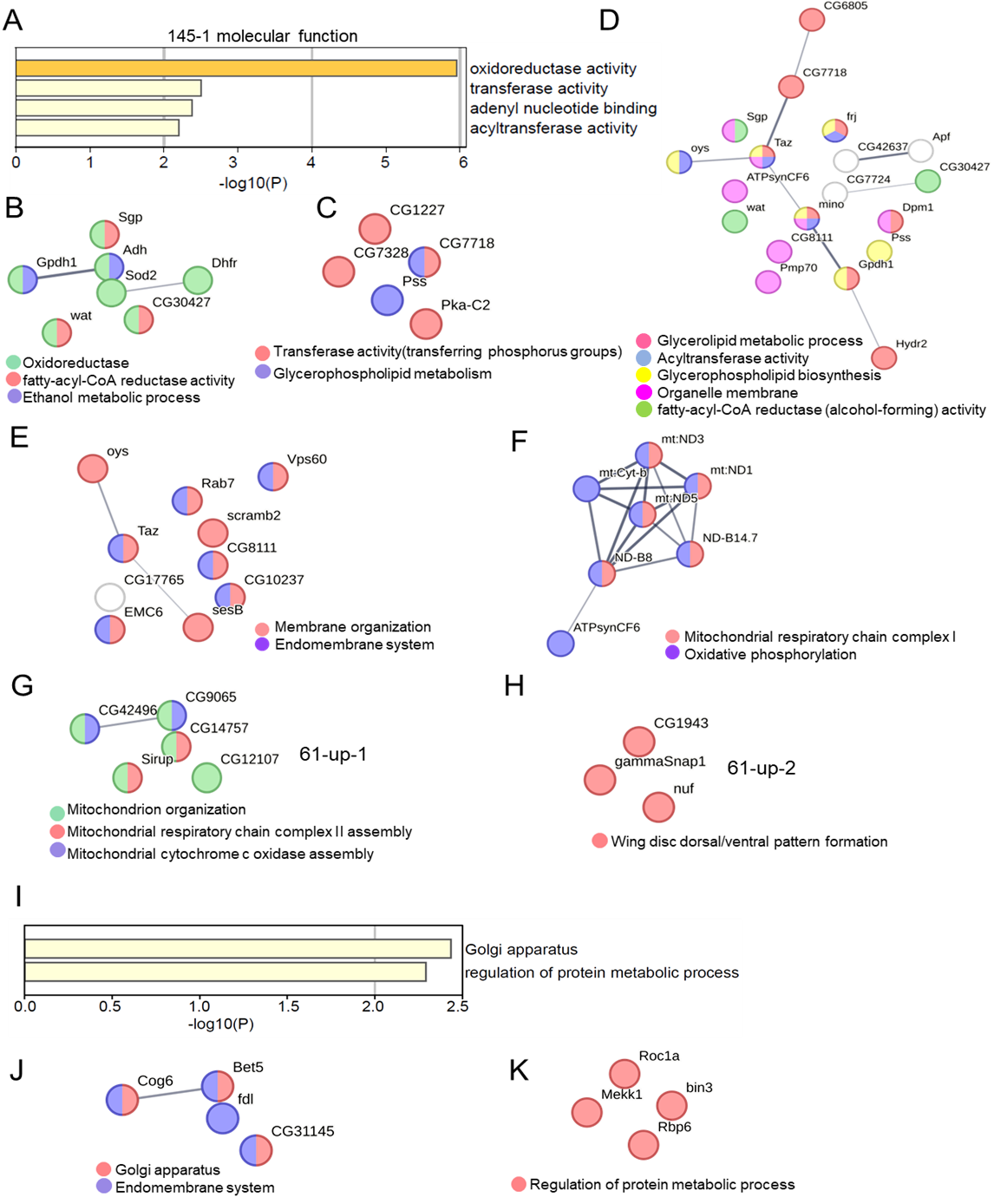
**Supplementary Figure 2. Analyzing the proteomes specifically down-regulated in aged flies and tissues**

**(A).** StringDB functional network plotting of the head-specifically down-regulated proteins in Figure 3E. **(B).** KEGG and GO functional enrichment analysis of the thoracic proteomes involved in protein localization (presented in Figure 3F). **(C)**. KEGG and GO functional enrichment analysis of the molecular function of the thoracic proteins that are invovlved in cellular macromolecule localization (presented in Figure S2B). **(D-G)** StringDB functional network plotting of the thoracic proteins in Figure S2C **(D).** the proteins carry the GTPase activity, **(E)**. the protein carries the molecular carrier activity, **(F)**. the proteins involved in signal sequence binding, **(G)**. proteins carry the lipid transporter activity. **(H)**. StringDB functional network plotting of the thoracic proteins involved in mitochondrial translation (presented in Figure 3F). **(I)**. KEGG and GO functional enrichment analysis of the molecular function of the thoracic proteins involved in the regulation of transport in Figure 3F. **(J)**. KEGG and GO functional enrichment analysis of the molecular function of the whole body's proteins involved in the lipid metabolic process in Figure 3G. **(K-M)**. StringDB functional network plotting of the whole body's proteins involved in Figure S2J. **(K).** the proteins carry the lipase activity, **(L).** The proteins carry the phosphotransferase activity and **(M).** The proteins carry the acyltransferase activity. **(N).** KEGG and GO functional enrichment analysis of the molecular function of the whole body's proteins involved in the carbohydrate metabolic process (presented in Figure 3G). **(O, P)**. StringDB functional network plotting of the whole body's proteins involved in Figure S2N. **(O)**. proteins carry the hydrolase activity and **(P)** proteins involved in carbohydrate binding. **(Q)**. StringDB functional network plotting of the whole body's proteins invloved in insemination in Figure 3G.

**
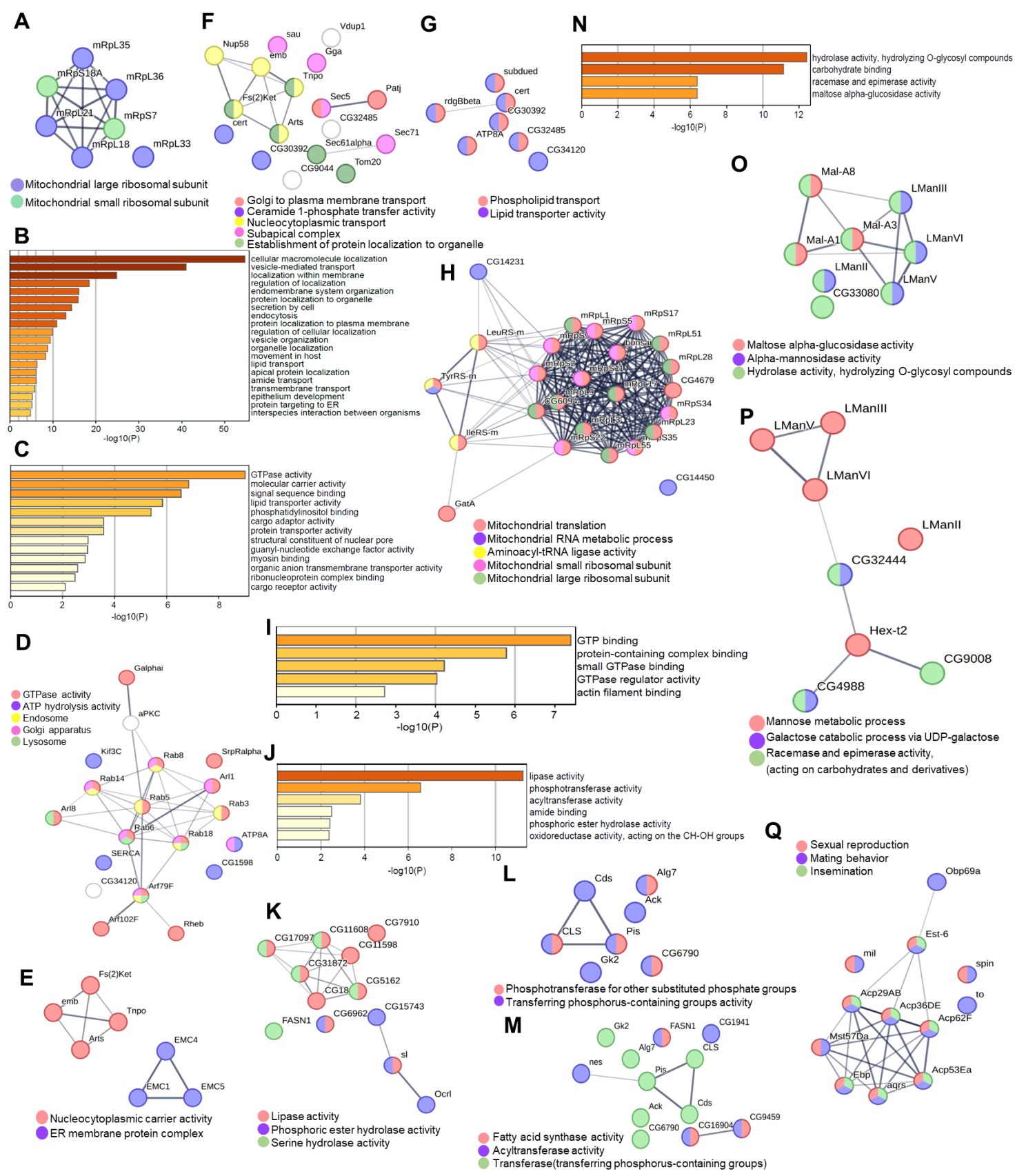
****Supplementary Figure 3. Analyzing the proteomes specifically up-regulated in aged flies and tissues**

**(A-D).** StringDB functional network plotting of the specifically up-regulated thoracic proteome in Figure 3H. **(A)**. proteins involved in cytoplasmic translation, **(B)**. proteins involved in regulating protein folding, **(C)**. proteins involved in the ubiquinone metabolic process, **(D)**. proteins involved in mitochondrion organization. **(E-G)**. StringDB functional network plotting of the up-regulated whole bodies’ proteome in Figure 3I. **(E)**. proteins involved in positive regulation of anti-fungal peptide production, **(F)**. proteins involved in the regulation of G protein-coupled receptor signaling pathway, **(G)**. proteins involved in mRNA processing. **(H-J)**. StringDB functional network plotting of the specifically up-regulated midguts proteome in Figure 3J. **(H)**. proteins involved in sperm storage, **(I)**. proteins involved in the lipid metabolic process, **(J)**. proteins involved in the glutathione metabolic process.

**
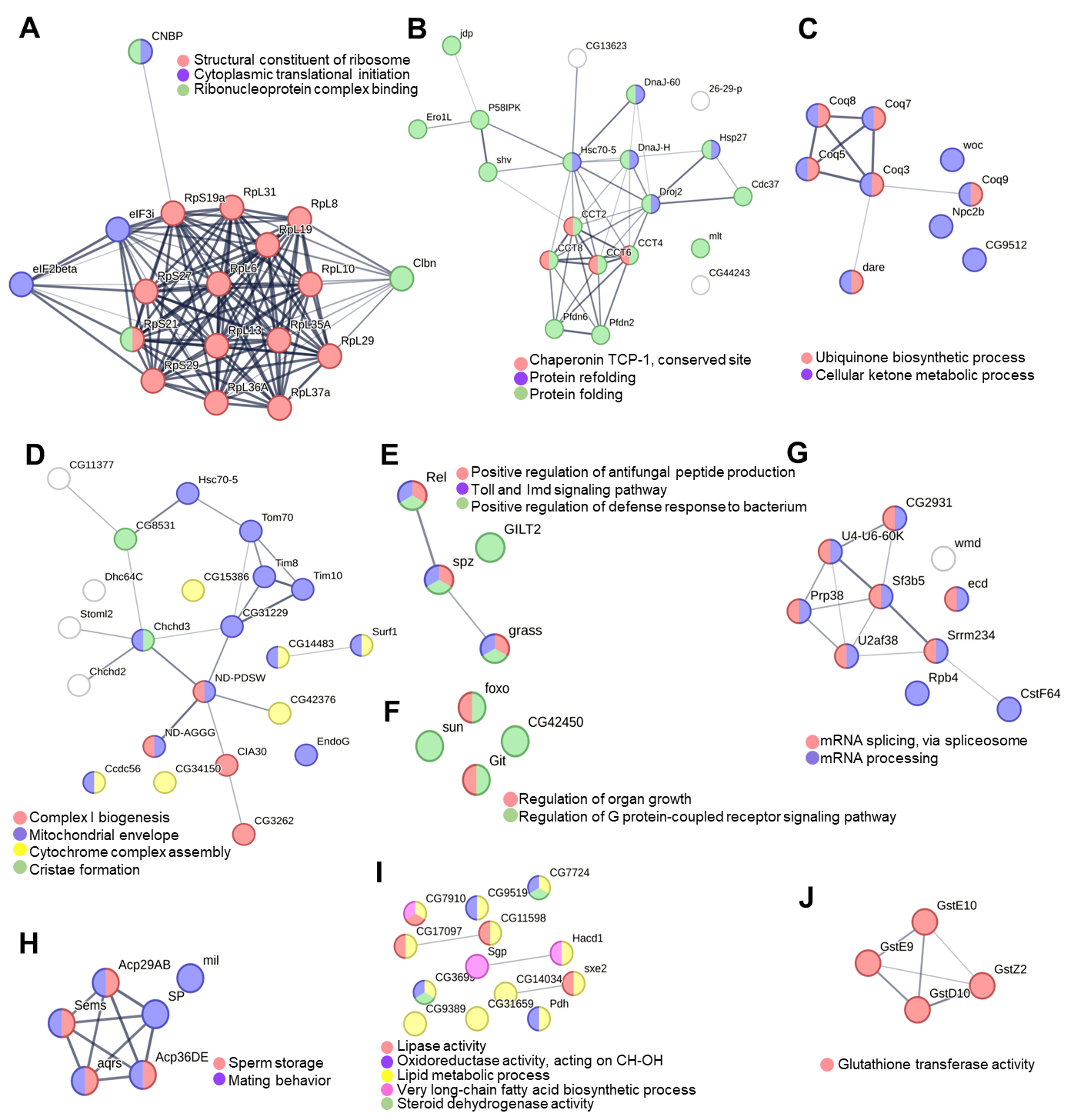
**

**Supplementary Figure 4. Ageing-associated degeneration of the midgut barrier**

**(A).** The Smurf assay is employed for the assessment of midgut permeability, wherein the permeability of a food blue dye is observed. The quantification of the ratio of flies exhibiting the Smurf phenotype (including mild and prominent Smurf) during the aging process is presented in **(B)**. ***: *p*<0.001. **(C)**. The relative content of food blue dye in the aged fly body is quantified by measuring its intensity. **: *p*<0.01. **(D)**. Permeability of 70 KD FITC-Dextran is observed in fly midguts. Scale bar=20 μm. **(E)** is the quantitative result of **(D)**, ***: *p*<0.001.


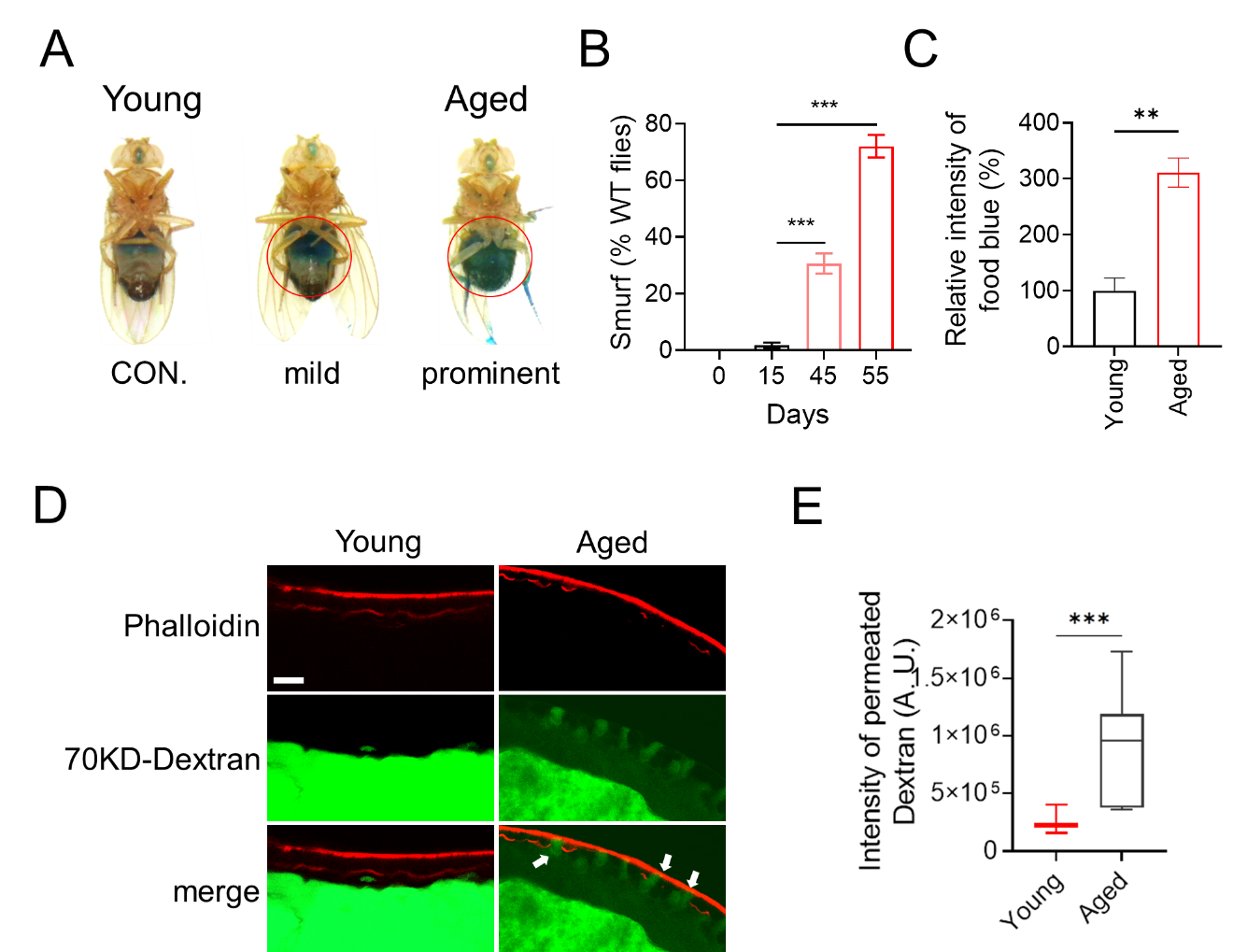


**Supplementary Figure 5. Insulin signaling in aged midguts**

**(A, B)**. The expression levels of *Thor* and *tobi* in aged midguts are analyzed by quantitative PCR. *rp49* is used as the internal control. ***: *p*<0.001, ****: *p*<0.0001. **(C, D)**. The levels of p-AKT and AKT are analyzed by immunoblot. *: *p*<0.05. Tubulin is used as the loading control. **(E)**. The efficiency of InR RNAi is evaluated by quantitative PCR. Da-Gal4 is used to drive the ubiquitous InR RNAi. MHC-Gal4 is used to drive InR RNAi in fly indirect flight muscles. Elav-Gal4 is used to drive InR RNAi in fly CNS. NP1-Gal4 is used to drive InR RNAi in fly midgut enterocytes. *rp49* is used as the internal reference. ***: *p*<0.001, ****: *p*<0.0001.

**
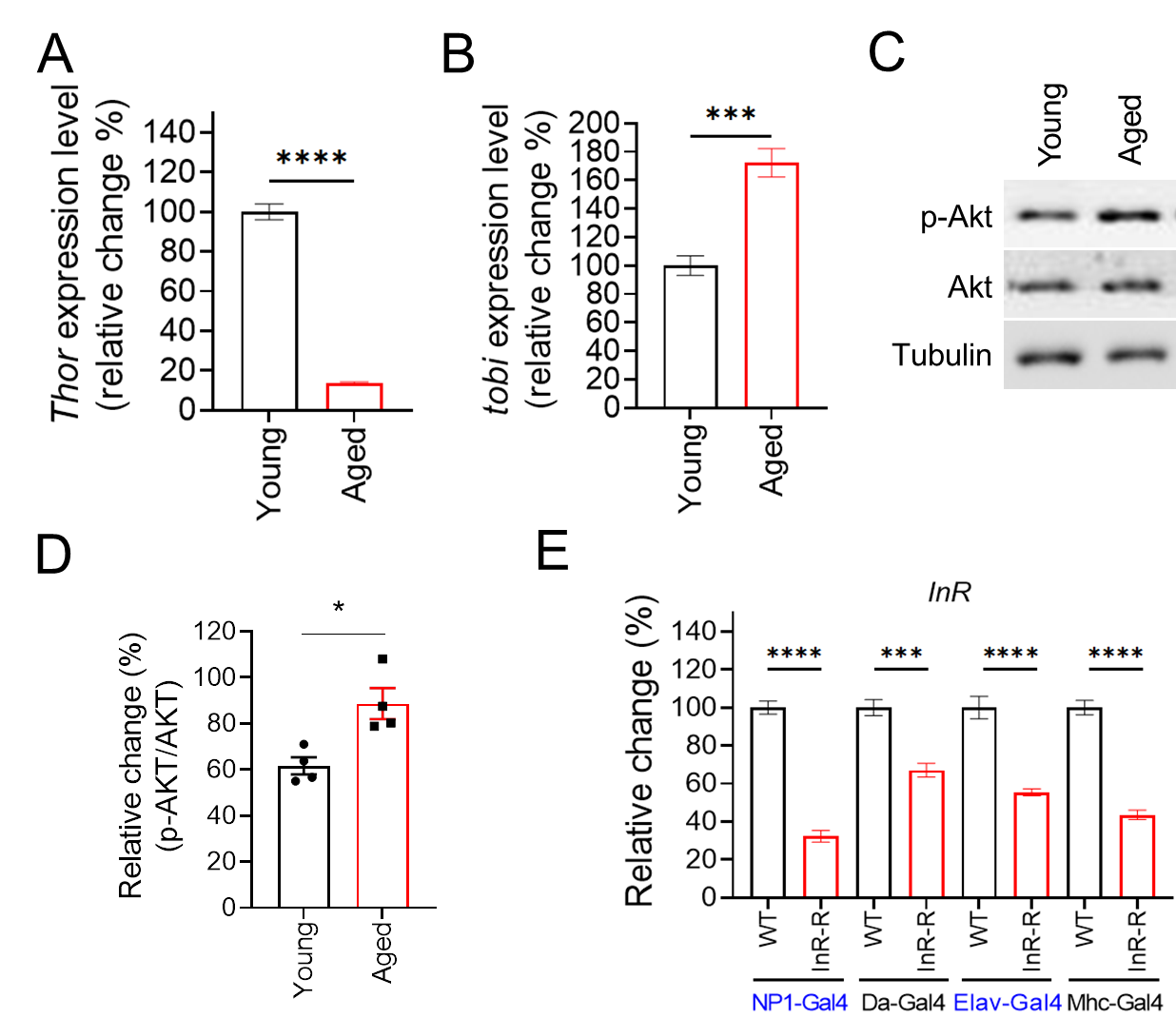
**

**Supplementary Figure 6. Analyzing the common signature between young flies and InR RNAi flies**

**(A, B)**. Overlap analysis of proteins down\up-regulated in the heads of young flies and flies with CNS-specific InR RNAi. The number of common and tissue specifically down\up-regulated proteins are presented in the circle areas. The one commonly up-regulated protein CG8736 is indicated. **(C, D)**. Overlap analysis of the proteins down\up-regulated in the thoraxes of young flies and flies with muscle-specific InR RNAi. The number of common and tissue specifically down\up-regulated proteins are presented in the circle areas. The four commonly up-regulated proteins ND-B8, RagC-D, CG6984 and Obp57a are indicated. **(E, F).** Overlap analysis of proteins down\up-regulated in midguts of young flies and flies with midgut-specific InR RNAi. The number of commonly and tissue-specifically down-regulated proteins are presented in the circle areas. **(G)**. KEGG and GO functional enrichment analysis of the commonly down-regulated proteins in midguts that indicated in **(E)**. **(H)**. StringDB functional network plotting of the specifically down-regulated proteins involved in sperm storage, metabolism of steroid hormones and alpha-amino acid catabolic process.

**
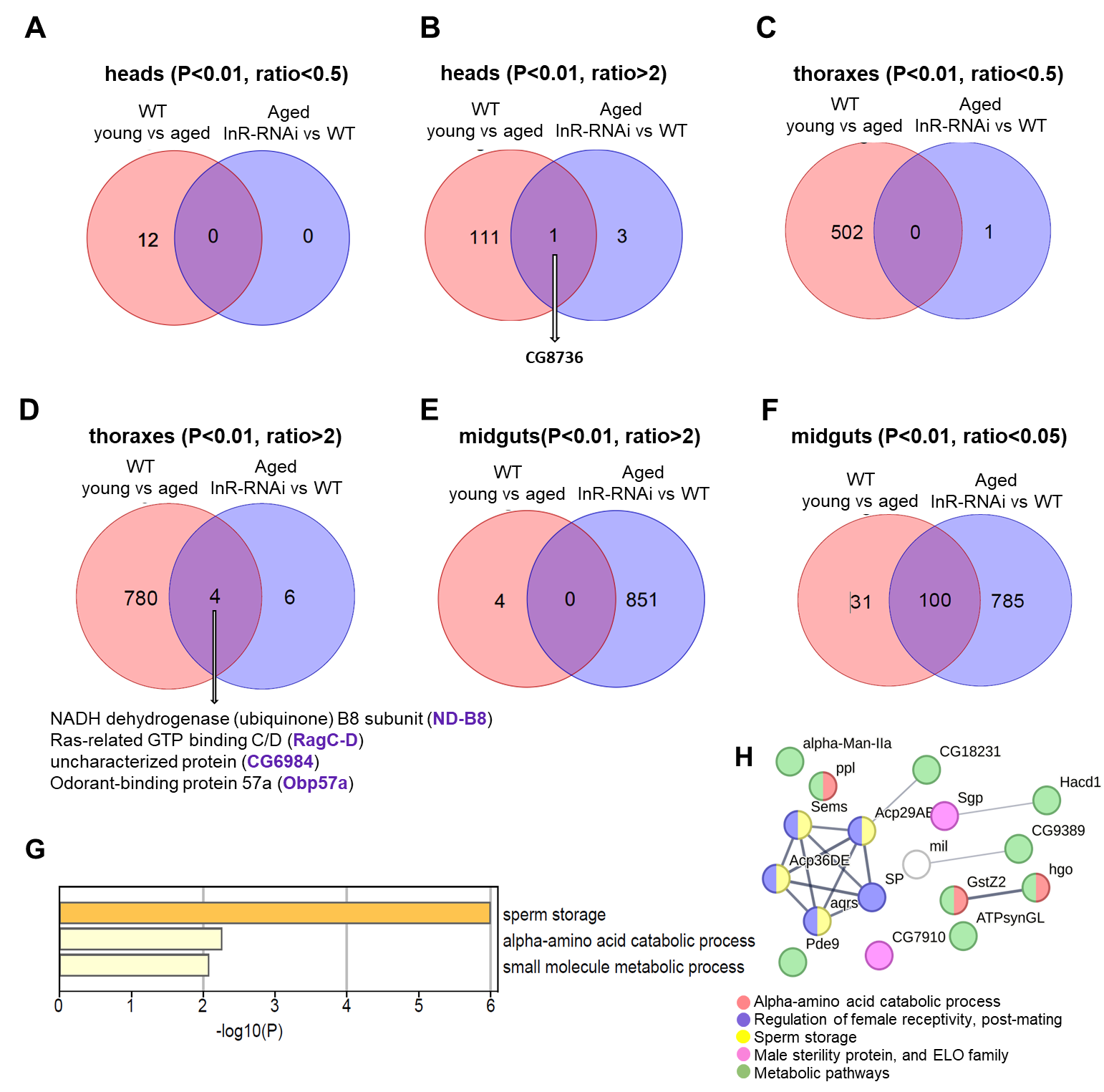
**

**Supplementary Figure 7. Degeneration of the midgut actin network during aging**

**(A).** Aged midguts are stained with phalloidin-TRITC to visualize the Actin network. Scale bar=20 μm. **(B)** is the quantitative result of **(A)**, ***: *p*<0.001. **(C)**. The relative content of food blue dye in flies is assessed by quantifying the intensity of the dye. *: *p*<0.05; ****: *p*<0.0001. **(D)**. Permeability of 70 KD FITC-Dextran is observed in young fly midguts. NP1-Gal4 is used to drive the knocking-down of InR in midgut enterocytes. Scale bar=20 μm. **(E)** is the quantitative result of **(D)**. **(F)**. Survival curves of young WT and midgut-specific InR RNAi flies during *Staphylococcus aureus* infection.


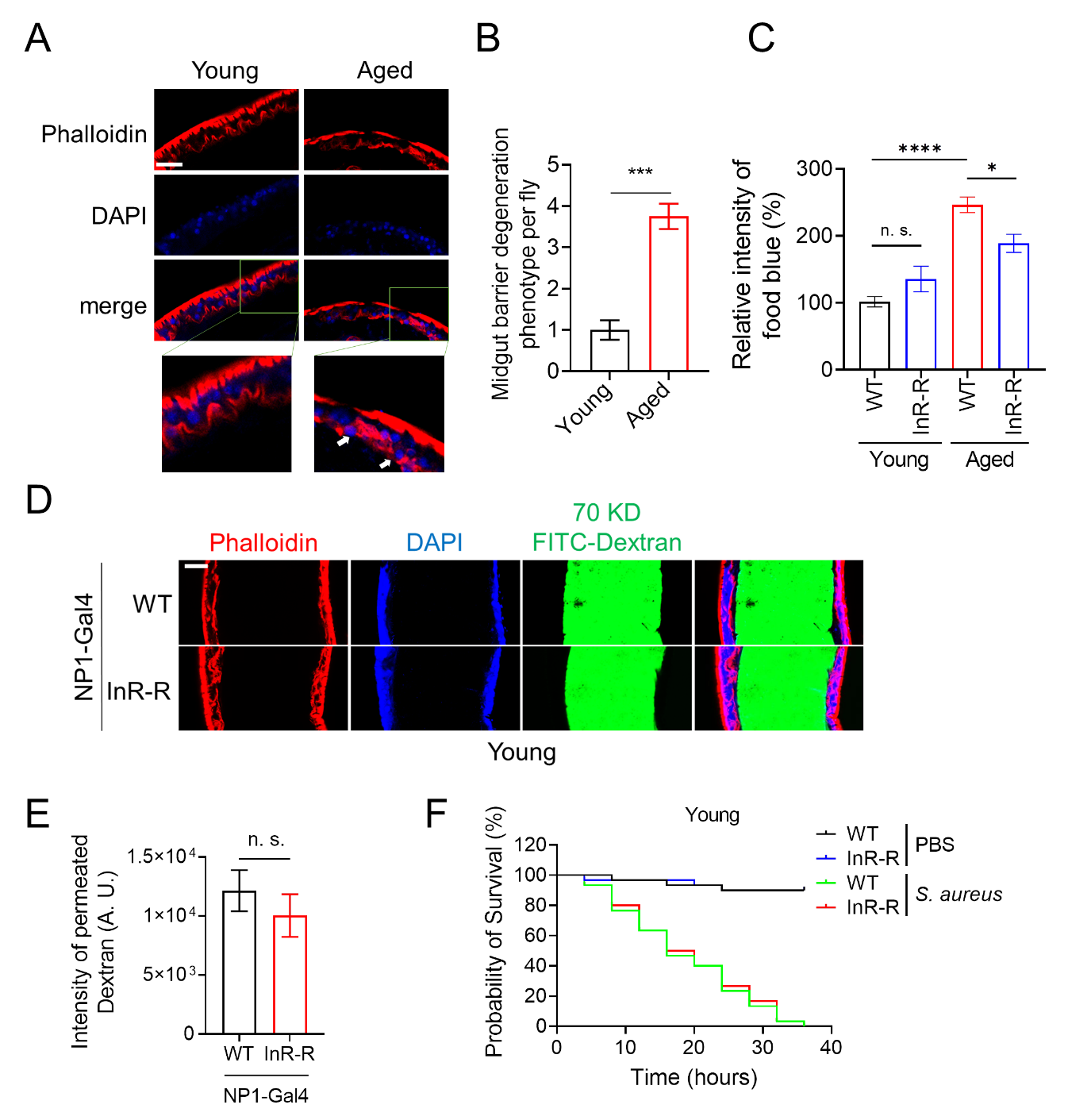


**Supplementary Figure 8. Down-regulation of InR in midgut delays the functional decline of gut cells**

**(A, B).** The expression levels of PGRP-LC and PGRP-SD in young and aged flies are analyzed using quantitative PCR (qPCR). *: *p*<0.05; ***: *p*<0.001; ****: *p*<0.0001. NP1-Gal4 is utilized to drive the knockdown of InR specifically in the gut epithelial cells of flies. **(C, D).** Immunostaining of Phospho-Histone H3 is performed on fly midguts. The scale bar represents 50 μm. NP1-Gal4 is used to induce knockdown of InR in the gut epithelial cells of flies. **(D)** is the quantitative results of (C). ****: *p*<0.0001. **(E).** Tetramethylrhodamine ethyl ester perchlorate (TMRE) staining is conducted on fly midguts. Scale bar= 50 μm. NP1-Gal4 is employed to drive the knockdown of InR specifically in the gut epithelial cells of flies. **(F)** is the quantitative result of **(E).** ****: *p*<0.0001. **(G).** The levels of ubiquitinated proteins and ref(2)P of aged fly midguts are analyzed via immunoblot. Tubulin is used as the loading control. **(H).** is the quantitative result of (G). **: *p*<0.01; ***: *p*<0.001. **(I).** The climbing ability of aged flies with midgut-specific InR knockdown after approximately 50 days aging period. NP1-Gal4 drives RNA interference targeting InR specifically in midgut enterocytes. ****: *p*<0.0001.


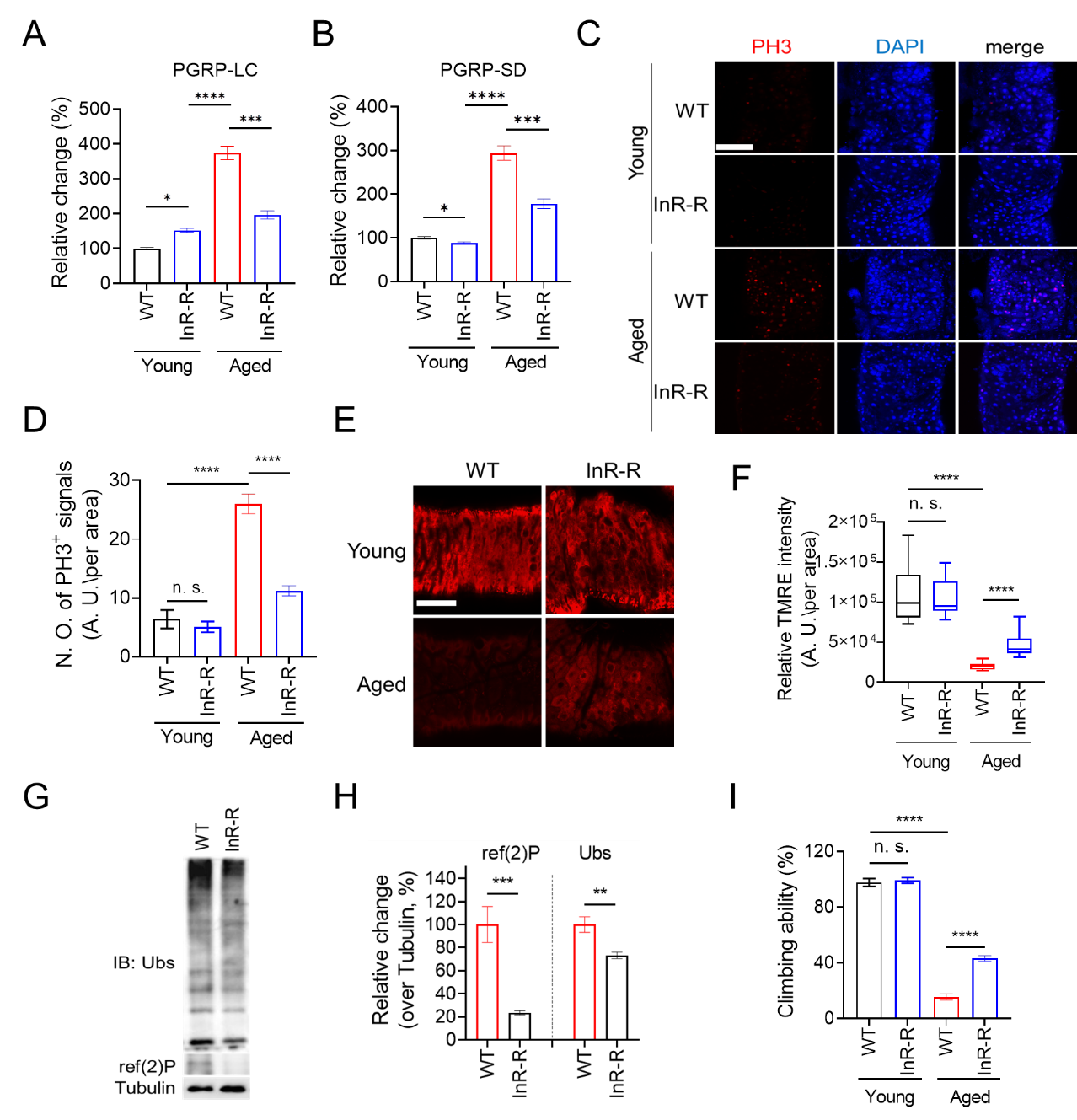

Supplement: Supplementary file 1 — Data S1: Supporting Information. [file ACEL-24-e14344-s001.docx]
